# Genetic basis of qualitative and quantitative resistance to powdery mildew in wheat: from consensus regions to candidate genes

**DOI:** 10.1186/1471-2164-14-562

**Published:** 2013-08-19

**Authors:** Daniela Marone, Maria A Russo, Giovanni Laidò, Pasquale De Vita, Roberto Papa, Antonio Blanco, Agata Gadaleta, Diego Rubiales, Anna M Mastrangelo

**Affiliations:** 1 Consiglio per la Ricerca e la Sperimentazione in Agricoltura - Cereal Research Centre, SS 673 km 25.2, Foggia 71122, Italy; 2 Department of Agro-Forestry and Environmental Biology and Chemistry, University of Bari, Via Amendola, 165/A, Bari 70126, Italy; 3 Institute for Sustainable Agriculture, CSIC, Apdo. 4084, Córdoba 14080, Spain

**Keywords:** Wheat, Powdery mildew, MQTL, Collinearity, Resistance gene

## Abstract

**Background:**

Powdery mildew (*Blumeria graminis* f. sp. *tritici*) is one of the most damaging diseases of wheat. The objective of this study was to identify the wheat genomic regions that are involved in the control of powdery mildew resistance through a quantitative trait loci (QTL) meta-analysis approach. This meta-analysis allows the use of collected QTL data from different published studies to obtain consensus QTL across different genetic backgrounds, thus providing a better definition of the regions responsible for the trait, and the possibility to obtain molecular markers that will be suitable for marker-assisted selection.

**Results:**

Five QTL for resistance to powdery mildew were identified under field conditions in the durum-wheat segregating population Creso × Pedroso. An integrated map was developed for the projection of resistance genes/ alleles and the QTL from the present study and the literature, and to investigate their distribution in the wheat genome. Molecular markers that correspond to candidate genes for plant responses to pathogens were also projected onto the map, particularly considering NBS-LRR and receptor-like protein kinases. More than 80 independent QTL and 51 resistance genes from 62 different mapping populations were projected onto the consensus map using the Biomercator statistical software. Twenty-four MQTL that comprised 2–6 initial QTL that had widely varying confidence intervals were found on 15 chromosomes. The co-location of the resistance QTL and genes was investigated. Moreover, from analysis of the sequences of DArT markers, 28 DArT clones mapped on wheat chromosomes have been shown to be associated with the NBS-LRR genes and positioned in the same regions as the MQTL for powdery mildew resistance.

**Conclusions:**

The results from the present study provide a detailed analysis of the genetic basis of resistance to powdery mildew in wheat. The study of the Creso × Pedroso durum-wheat population has revealed some QTL that had not been previously identified. Furthermore, the analysis of the co-localization of resistance loci and functional markers provides a large list of candidate genes and opens up a new perspective for the fine mapping and isolation of resistance genes, and for the marker-assisted improvement of resistance in wheat.

## Background

Bread wheat (*Triticum aestivum* L.) and durum wheat [*Triticum turgidum* (L.) subsp. *turgidum* (L.) convar. *durum* (Desf.)] are among the most important cultivated crops worldwide. Powdery mildew, which is caused by the fungus *Blumeria graminis* f.sp *tritici*, results in important yield losses and affects stable wheat production in areas with cool or maritime climates. Breeding of resistant cultivars is the most economical and environmentally sound method to decrease the use of fungicides and to reduce crop losses due to this disease.

Both qualitatively and quantitatively inherited resistances have been reported in wheat against powdery mildew. Qualitative resistance to powdery mildew is controlled by major race-specific genes that are effective only against some isolates of powdery mildew. Unfortunately, qualitative resistance is usually of short durability, due to frequent changes in the pathogen population [1]. Consequently, new resistance genes are continuously needed to replace the defeated ones.

To date, more than 60 powdery mildew resistance genes/ alleles have been reported in common and durum wheat [2]. Some of these were transferred from domesticated as well as wild relatives, such as *Triticum turgidum* var. *dicoccoides* (Körn.) and var. *dicoccum* (Schrank), *T. timopheevii* (Zhuk.), *T. monococcum* (L.), *T. tauschii* (Schmalh), and *Aegilops speltoides* (Tausch), or from more distant species, like *Secale cereale* (L.) [2]. Molecular markers have largely been used for mapping a number of these genes to specific chromosomes or chromosome regions [3], and some of these genes have been cloned. In particular, *Pm3b* from hexaploid wheat is a member of the coiled-coil nucleotide binding site leucine-rich repeat (*NBS-LRR*) class of disease resistance genes, and it determines complete resistance [4]. Very often partial resistance is a polygenic trait, but this is not always the case. An example of monogenic partial resistance is the gene *Mlo*. Homologs of the barley [*Hordeum vulgare* (L.)] gene *Mlo* were found in syntenic positions in all three genomes of hexaploid wheat [5-8]. The *Mlo* gene was isolated by positional cloning, and its deduced amino-acid sequence revealed no homologies to other characterized plant R proteins [9]. This sequence defines an integral membrane protein with seven transmembrane helices and two casein kinase II motifs. A putative serine/ threonine protein kinase gene (*Stpk-V*) in the *Pm21* locus was also characterized as conferring durable resistance; it is located on chromosome 6 V of *Haynaldia villosa* (L.) and was transferred to wheat as a 6VS · 6AL translocation [10].

The polygenic nature of partial resistance to powdery mildew makes it more complicated to handle in any breeding programs, compared to race-specific resistance. Many reports on high-density linkage maps and quantitative trait loci (QTL) that govern this trait are available in the literature, e.g., [11-13]. These QTL have been mapped against a single genetic background, and they have been evaluated in a limited number of environments. Moreover, it is difficult to predict the usefulness of QTL for marker-assisted selection based only on the QTL performance in an individual genetic background in any particular study. Goffinet and Gerber [14] proposed a method to combine the results from independent studies, called QTL meta-analysis, which allows the numbers and consensus positions of ‘real’ QTL involved in the control of a certain trait to be obtained. These QTL regions from the QTL meta-analysis (‘meta-QTL’, or MQTL) have refined, or consensus, positions, and they can facilitate the identification of positional candidate genes. In wheat, some examples of MQTL have been reported. Quraishi et al. [15] provided an overall view of MQTL for nitrogen use efficiency and grain dietary fiber content in bread wheat, with an unraveling of the candidate genes for these traits. Stable QTL that have provided MQTL have been defined also for yield, yield components, and crop height, which are very important traits in wheat breeding [3,16]. Ear emergence and resistance against *Fusarium* head blight have also been studied in wheat, which resulted in increased precision in the QTL position estimations and the identification of molecular markers linked to them [17-20].

In the present study, we report on the MQTL for resistance to powdery mildew in wheat. The objectives were: 1) to identify new sources for resistance to powdery mildew in durum wheat; 2) to develop a high-density integrated map for the projection of resistance genes/ alleles and QTL, and to investigate their distribution in the wheat genome; 3) to identify MQTL across studies to validate results from independent studies and reliable markers for marker-assisted selection; and 4) to identify the homologous regions of MQTL in other crops using a comparative genomics approach, to provide candidate genes that drive this trait.

## Results and discussion

### QTL mapping for resistance to powdery mildew in the durum-wheat Creso × Pedroso RIL population

The durum-wheat cultivars Creso and Pedroso have been evaluated for different traits and have shown some interesting phenotypes, in particular for disease resistance. A major QTL for resistance to leaf rust was previously mapped on the long arm of chromosome 7B of Creso [21]. In the present study, we performed a combined ANOVA in the recombinant inbred line (RIL) population for powdery mildew disease severity over two environments. The mean squares due to RILs, environments, and RIL × environment interactions were highly significant. The means of the parental lines, the means and ranges of the RIL population, and the variance components estimated for each trait over the two environments are reported in Additional file 1. The parents of the segregating population showed a significant, although small, difference, with higher disease severity observed for Pedroso. This difference was consistent for both of the environments, although the infection pressure was higher in Italy than in Spain. The continuous phenotypic frequency distributions across the two environments of the 123 RILs for disease severity score showed a normal distribution, which suggests a quantitative genetic basis for this trait (data not shown).

A total of 5 QTL were detected in the present study, on chromosomes 6A, 2B, 3B and 6B, with logarithm of odds (LOD) values between 3.0 and 5.0 (Additional file 2). The observed variability for the phenotype ranged from 10.6% to 18.5%. Interestingly, the two QTL named CP 3 and located at 16 cM on chromosome 6B could correspond to the same genomic region, which would be common to both environments. This idea is supported because the two QTL fall within the same region on the integrated map (Additional file 3: Figure S1). All of the other QTL identified in the present study were expressed only in one environment, probably due to differences in the natural populations of powdery mildew present in the two environments. The alleles of resistance to powdery mildew were contributed by both parents. In particular, based on the sign of the additive effects, the allele of Creso was effective in decreasing disease severity for QTL CP 2; for the other QTL, the resistant phenotype was contributed by the allele of Pedroso. The limited difference in resistance between the two parents justifies the possibility that some resistant alleles are also contributed by the cv. Pedroso. Furthermore, the low disease pressure registered in the two environments can allow to detect a qualitative resistance more than a quantitative one. Another possible explanation of our results is that a major QTL or other QTL contributed by the cv. Creso could be present in genomic regions that are not covered by molecular markers in the Creso x Pedroso genetic map. In this case they have not been detected in the present study, while only minor QTL are evident. The map positions of the QTL are reported in Additional file 3: Figure S1, except for QTL CP 4, for which the projection was not possible, as only one marker (*Xbarc134* - 121.6 cM) was in common between the Creso × Pedroso map and the integrated map developed in the present study. As will be described in detail in the following paragraphs, the QTL CP 2, 3, 4, and 5 did not fall into MQTL regions, even if they were at a distance of <10 cM from other individual QTL. For this reason we cannot be sure that they correspond to regions not previously identified. Interestingly, QTL CP 1 on chromosome 6A identified a genomic region that is characterized by the absence of previously described QTL. Therefore, the results from the analysis of the Creso × Pedroso population have revealed a new source of quantitatively inherited resistance to powdery mildew.

### Development of a wheat integrated map

A dense consensus map was obtained by merging the recent durum-wheat high-density consensus map developed by Marone et al. [22] with two well-saturated bread-wheat maps [23,24]. The resulting integrated map represents a useful tool for MQTL, by starting from genetic loci that were identified in both tetraploid and hexaploid wheat. After that, a number of partial genetic maps related to small chromosomal regions were projected onto the integrated map; these regions contained genes/ QTL of resistance to powdery mildew, as revealed by the present study and previous studies. Furthermore, as a large proportion of diversity arrays technology (DArT) markers corresponding to expressed sequences was revealed to code for resistance proteins, such as NBS–LRR proteins or LRR kinases [25], the chromosomal regions from the genetic maps available in the literature and containing these DArT markers were also projected onto the map.

The final consensus map was composed of 3,618 markers (DArT, simple sequence repeat [SSR], expressed sequence tag [EST]-derived, sequence-tagged sites, and restriction fragment length polymorphism) and spanned a total map length of 3,723.9 cM (Table 1; Additional file 3: Figure S1; Additional file 4). A total of 1,260 markers were positioned on genome A, 1,876 on genome B, and only 482 on genome D, even if the total lengths were not very different (Table 1). The average distance between markers on the whole genome was 1 cM per marker, thus enabling the more precise location of the QTL.

**Table 1:** Summary of the main features of the wheat integrated map

**Chromosome**	**N° anonymous markers**	**N° markers corresponding to NBS-LRR/KIN**	**N° markers with other putative function**	**Total markers**	**Length (cM)**	**Density (cM/marker)**
1A	132	2/0	8	142	108.3	0.7
2A	159	6/1	2	168	98.2	0.6
3A	119	1/0	13	133	167.8	0.8
4A	234	10/1	18	263	207.1	0.8
5A	120	1/0	15	136	209.4	1.5
6A	208	3/2	3	216	206.5	0.9
7A	177	8/5	12	202	260.5	1.3
Genome A	1,149	31/9	71	1,260	1,257.8	1.0
1B	264	3/3	9	279	224.8	0.8
2B	267	9/4	6	286	205.9	0.7
3B	360	6/3	3	372	138.5	0.4
4B	107	0/1	5	113	153.1	1.3
5B	184	2/3	7	196	278.9	1.4
6B	267	8/2	7	284	180.8	0.6
7B	317	5/4	20	346	238.9	0.7
Genome B	1,766	33/20	57	1,876	1,420.9	0.8
1D	103	3/1	1	108	133.1	1.2
2D	79	1/1	0	81	126.6	0.6
3D	59	0/0	0	59	291.9	4.9
4D	37	0/0	0	37	128.6	3.5
5D	83	0/0	1	84	101	1.2
6D	35	2/0	0	37	110	3
7D	73	3/0	0	76	154	2
Genome D	469	9/2	2	482	1,045.2	2.2
Genomes A, B, D	3,384	73/31	130	3,618	3,723.9	1.0

*cM* centimorgan.

Out of 3,618 markers mapped, 295 corresponded to putatively expressed sequences, and for most of these (234), a putative function could be proposed based on similarity searches (Additional file 4). In many cases, this function was related to plant responses to diseases. Also in this case, the A and B genomes were much more represented than the D genome. In particular, one PCR-based molecular marker was found to correspond to a *NBS-LRR* gene (*Xcdo244* on 2B - 129.4 cM), and five other markers to three protein kinases (*TC91851* on 5A - 85.4 cM -, *Xbcd1088* on 5A - 95 cM -, and *Xmag1759* on 7A - 206.6 cM) and two pathogenesis-related proteins (*TC77302* on 3A - 151.8 cM - and *TC92445* on 7A - 226.9 cM). Finally, out of 105 mapped DArT markers, 72 corresponded to NBS-LRR proteins (Additional file 3: Figure S1, purple) and 33 to protein kinases, with most of these containing a LRR domain (Additional file 3: Figure S1, brown). These markers were spread all over the genome, except for chromosomes 3D, 4B, 4D, and 5D. The number per chromosome was from 1 (3A and 5A) to 13 (2B and 7A). Eleven of the *NBS-LRR* genes showed similarity with proteins belonging to the subgroup of coiled-coil *NBS-LRR* genes (*wPt-1862* on chromosome 1A - 45.1 cM -, *wPt-6059* - 72.6 cM - and *wPt-7421* - 81.9 cM - on chromosome 1D, *wPt-6064* on chromosome 2A - 80.3 cM -, *wPt-4368* on chromosome 2B - 118.7 cM -, *wPt-4077* on chromosome 3A - 82.5 cM -, *wPt-1516* - 11.7 cM - and *wPt-8845* - 114.6 cM - on chromosome 3B, *wPt-3729* on chromosome 4A - 177.6 cM -, *wPt-4936* on chromosome 5B - 120.8 cM -, and *wPt-0833* on chromosome 7D - 16.3 cM). Furthermore, four DArT markers were similar to *NBS-LRR* and also contained an additional BED zinc finger domain (*wPt-2120* on 2B - 73.3 cM -, *wPt-0950* on 2B - 93 cM -, *wPt-0189* on 2B - 113.3 cM -, and *wPt-4660* on 4A - 73.7 cM).

The sequences of the markers corresponding to *R* genes were searched against the database of the wheat separate chromosome arms promoted by the International Wheat Genome Sequencing Consortium (http://urgi.versailles.inra.fr/srs83/displayTool.do?toolName=BlastN), to study other genes eventually present in the same genomic clone. The results of the searches are reported in Additional file 5. In many cases, other genes were present, and in particular, genes corresponding to transposons and retrotransposons, exonucleases, acyl transferase, RNaseH, and other unknown proteins. In particular, transposons are recognized as one of the factors that drive gene duplication and amplification events that are at the basis of the evolution of this class of genes [26]. However, in other cases, more copies of resistance genes were observed. The marker *wPt-4077* (82.5 cM) corresponded to a genomic region on the long arm of chromosome 3A that contained two copies in tandem (at a distance of about 2000 bp) and in the same orientation as a *NBS-LRR* gene similar to the Rp1-like gene of *Brachypodium distachion*. There were also two copies of a *NBS-LRR* gene in the region of chromosome 4A, where the marker *wPt-6303* (55.6 cM) was positioned. In this case, the sequences were similar to a gene of *Zea mays*, and they showed opposite orientation. The distance was again *ca*. 2000 bp. Similar cases were observed for the markers *wPt-0992a* (176.9 cM) and *wPt-3729* (177.6 cM) on chromosome 4A, and *wPt-2782* (83.4 cM) on chromosome 6D. Two genes coding for receptor-like kinases were found within the genomic clone corresponding to the marker *wPt-8238* (70.6 cM) on chromosome 3B.

In some cases, three copies of a gene were found in the clone. This was the case for the markers *wPt-1314* (chromosome 6D - 90.4 cM), which corresponds to a *NBS-LRR* gene, and *wPt-5011* (chromosome 1B - 59.1 cM), which corresponds to a LRR receptor-like protein. For this marker, the genomic clone on chromosome 1B contained three genes, the first in opposite orientation with respect to the second and third ones. There was also a genomic clone in a homoelogous position on the long arm of chromosome 1A, and also in this case there were three copies of the gene, with the same orientation as the corresponding genes in the homoeologous locus.

In other cases, clusters of resistance genes of different types were observed. The marker *wPt-2147* (44.8 cM) on the short arm of chromosome 2A fell into a region containing a *NBS-LRR* gene and a gene coding for a probable receptor-like kinase. On the same chromosome, the marker *wPt-1601a* (48 cM) corresponded to a genomic clone that contained a protein kinase and a *NBS-LRR* gene. *R* genes are organized in large families that are subjected to continuous evolution, because of their constant interactions with the evolving pathogens. They often occur in clusters at specific loci following gene duplication and amplification events, and these clusters can combine similar or different genes, as has been shown for a number of species [27]. The results reported in the present study confirm this feature also in wheat.

An abundance of *R* genes in plant genomes has been described in many species. As an example, around 600 *R* gene loci were identified by Shang et al. [28] in a genome-wide comparison of the major class of *R* genes between the indica and japonica rice varieties. Knowledge of the map positions of these genes is of great importance for genetic studies that are aimed at the identification and fine mapping of resistance determinants. Furthermore, positioning these genes in genetic maps rich in molecular markers offers the chance to select many molecular markers closely linked to resistance determinants, to be used in marker-assisted selection programs [27]. Forty DArT sequences that correspond directly or indirectly to *NBS-LRR* genes and protein kinases were mapped in durum wheat in a previous study, and many of these were positioned in regions where determinants of resistance to a number of plant pathogens have been identified [22]. The construction of a dense integrated map allowed us to extend the number of markers corresponding to *R* genes mapped on the wheat genome: a total of 117 genes were mapped (104 markers positioned on the map, plus 13 additional genes found in the same genomic clone of some markers), which comprised 79 *NBS-LRR* genes and 38 protein kinases, most of which contained LRR domains.

### Meta-analysis results

Meta-analysis provides the estimates of QTL consensus positions and the confidence intervals (CIs) where the causative polymorphisms of the QTL are most likely to be positioned [29]. MQTL have been used for different traits in wheat and other crops, such as for wheat-grain morphology [30], for digestibility and cell-wall traits in maize [29], for grain yield in rice [31] and for multiple disease resistance in barley [32].

In total, this analysis used 20 publications that described 96 QTL for powdery mildew resistance, plus the 5 QTL described in the present study (101 in total). The characteristics of the phenotyping experiments and mapping populations are reported in Additional file 6. The chromosomal locations of 52 resistance genes/ alleles collected from 48 different mapping populations are also reported in this study (Additional file 3: Figure S1; Additional file 7). Three additional genes for resistance to powdery mildew have been published over the last few months, and therefore they are not included in the present analysis, which was already finalized at that stage. The gene *Ml5323* has been mapped to chromosome 2B [33] in a region that corresponds to the gene *MlIW170* that was mapped in the present study. Moreover, another gene (*Pm46*) has been reported in the literature, in the same region as *Pm45*, under the individual QTL *QPm.inra-5D*[34]. The last gene, *Pm24b*, was reported as tightly associated to *Pm24* by Xue et al. [35], which was mapped in the present study on chromosome 1D.

The analysis resulted in 24 MQTL, each of which resulted from integration of at least two initial QTL. Thirty-seven out of the initial 101 QTL remained as singletons. The MQTL, along with their Akaike Information Criterion values, CIs, flanking markers, and number of initial QTL involved are reported in Table 2. The number of clustered initial QTL ranged from 2 to 6, whereas the 95% CI of the MQTL varied from 0.2 to 48.5 cM, with an average of 7.3 cM. MQTL were found on all chromosomes, except for 1D, 3B, 3D, 4D, 6B, and 6D, and their number per chromosome ranged from 1 to 3 (Additional file 3: Figure S1, Table 2).

**Table 2:** Characteristics of the MQTL identified in the present study

**MQTL**	**Chr**	**AIC value**	**Position (cM)**	**Mean initial CI (cM)**	**MQTL CI (95%) (cM)**	**Flanking markers**	**N° of initial QTL**	**N° of involved QTL**
MQTL1	1A	39	47.4	3.4	0.7	Xgdm33a-wPt-8072	8	3
MQTL2			56.3	14.4	1.3	Xcfd15a-Xgwm33		4
MQTL3	1B	40.2	17.9	30.1	9.7	wPt-3477-wPt-0655	5	2
MQTL4	2A	25.5	68.7	3.5	2.9	Xgwm47a-PmHNK54	7	2
MQTL5			85.7	11.5	0.6	XgbxG303-Xcfd267		3
MQTL6	2B	48.7	82.9	9	0.3	Xbarc98-Xbarc1147	8	2
MQTL7			100.1	6.4	1.5	Xcfd70-Xwmc149a		3
MQTL8			136.7	53.5	20.4	BJ253815-wPt-0471		2
MQTL9	2D	14.1	81	15.5	7.5	Xgwm157-Xcfd16	3	2
MQTL10	3A	20.3	42.4	67.9	48.5	Xcfd79a-Xwmc264a	2	2
MQTL11	4A	64.8	59.6	8.2	1.2	Xgwm111a-Xgwm894	8	2
MQTL12			146.8	73.6	10.5	Xbcd130-TC85050		3
MQTL13			174.8	20.8	7.1	wPt-1362-Xwmc104a		2
MQTL14	4B	13.7	109.3	14	3.9	Xbcd110-Xgwm6a	3	2
MQTL15	5A	93.2	89.6	43.7	3.4	Xbcd1355-Xbarc1	7	3
MQTL16			135.9	31.5	9.3	Xgwm443c-Xcfa2155		2
MQTL17	5B	51.5	105	20.3	8.3	Xbarc4a-wPt-1951	6	3
MQTL18	5D	38.4	47.7	3.9	1.7	PmY212-Xwmc818e	9	2
MQTL19			49.6	5.8	0.2	Xgwm174-Xwmc289		6
MQTL20	6A	12	129.9	15.5	3.8	Xwmc580-Xgwm617b	3	2
MQTL21	7A	21.6	112.5	41.7	16.9	Xwmc826d-wPt-3992	9	3
MQTL22		20.7	186.6	29.6	7.8	wPt-4553-NCA6Pm		3
MQTL23	7B	33.5	137.2	22.3	5.2	wPt-8938-PmTm4	5	3
MQTL24	7D	33.8	72.6	16.2	2	Xgpw1106-Lr34/Yr18/Pm	5	4

*Chr* chromosome, *AIC* Akaike Information Criterion, *CI* confidence interval, *cM* centimorgan.

In the present study, resistance ‘hot spots’ were highlighted, where the QTL detected in different studies are located within the same genomic region, together with the *Pm* genes, such as on chromosomes of groups 1, 2, 4, 5, and 7. The chromosomes that are richer in loci for resistance to powdery mildew are: chromosome 2B, with three MQTL, one individual QTL and 6 *Pm* genes, with two of these positioned under MQTL8; and chromosome 7A, with two MQTL, two individual QTL, and the highest number of *Pm* genes (13). No genes were mapped on chromosomes of group 4 and on 1B, 3A and 3D.

In more detail, the region comprised between MQTL1 and MQTL2 on chromosome 1A is characterized by the presence of four genes (*Pm3g*, *Pm3a*, *Pm3e*, and *Mlar*), one of which (*Pm3g*) is under MQTL1, and the others are a few cM from MQTL1 and MQTL2. On chromosome 2A, MQTL4 co-maps with *PmHNK54*, while *Pm4b* is under MQTL5. Four genes (*PmJM22*, *PmPS5B*, *MlAB10*, and *MlZec1*) are located in the region of MQTL8 on chromosome 2B, but only the first one co-maps. On chromosome 2D, the gene *Pm43* is localized near (1.4 cM from) MQTL9. Chromosome 5D has MQTL18, which includes *PmY212* and two other genes (*Pm35* and *PmY201*) that are mapped very close to MQTL19 (less than 1 cM away). The homoeologous group 7 is characterized by gene/ MQTL co-localization on all of the three chromosomes. MQTL22 on chromosome 7A includes *NCA6Pm*. Two genes (*Pm5d* and *PmTm4*) are under MQTL23 on chromosome 7B, and another two (*mlxbd* and *Pm5e*) are at very short distance from MQTL23. Finally, the gene *Lr34/Yr18/Pm* is included in MQTL24 on chromosome 7D.

Genes mapped at very small distances can represent allelic series at the same locus, or clusters of *R* genes. The three *Pm3* genes on chromosome 1A might represent an allelic series at the *Pm3* locus. Similar cases are *Pm4* (chromosome 2A) with three alleles, and *Pm5* (chromosome 7B) with two alleles. On chromosome 7A, two genes, *Mlm80* and *Mlm2033*, are mapped at a distance of 0.7 cM from each other on the consensus map, and Yao et al. [36] described these as two alleles of the same locus; however, in other cases, allelism tests might be necessary to indicate either different alleles at the same locus or tightly linked resistance genes, even with different resistance specificities. Schweizer and Stein [32] recently reported that loci for resistance to different diseases can associate in the same locus in barley. We found similar results in wheat, as we can identify the regions on chromosomes 4B and 4D that correspond to *TaMlo* loci, homologs of barley *Mlo*, based on common markers between our map and that developed by the International Triticeae Mapping Initiative. In particular, these correspond to the MQTL14 region on chromosome 4B and the single QTL *QPm.caas* on chromosome 4D. For chromosome 4A, two DArT markers that correspond to the barley *NBS2-RDG2a* gene for resistance to leaf stripe (*wPt-0992* - 176.9 cM ; [25]) and to a rust resistance Rp1-like protein of *T. aestivum* (*wPt-3729* - 177.6 cM ; [25]) are positioned in the region corresponding to MQTL13 on chromosome 4A. The *Pm1* gene is associated with the leaf and stem rust resistance genes *Lr20* and *Sr15* on chromosome 7A [37]. Many genes/ alleles are also located under or very close to a single QTL, as for chromosomes 1A, 1D, 2A, 3B, 5A, 5B, 5D, 6A, 6B, 6D, 7A, and 7B (Additional file 3: Figure S1). Resistance determinants are generally located in the telomeric and sub-telomeric regions, according to Schmolke et al. [38].

The chromosomal regions involved in the control of powdery mildew resistance were also investigated to find candidate genes for this trait, by searching in public databases for possible functions for these sequenced markers that putatively correspond to expressed genes. Many functional categories were identified, as annotated in Additional file 4. Most of the candidate genes encode NBS-LRR proteins, LRR-protein kinases, receptor-like protein kinases, WRKY or NAC transcription factors, pathogenesis-related proteins, cell transporters, or glutathione transferases (involved in the metabolism of reactive oxygen species), or proteins involved in lipid metabolism, amino-acid biosynthesis or cell wall modifications upon powdery mildew attack. As examples, on chromosome 1A there are genes that correspond to putative ketol-acid reductoisomerase, an enzyme induced by plant–pathogen interactions [39] near MQTL2 (*Xcdo1160* - 53.6 cM) and under the individual QTL QPm_Lan (*Xmwg632 -* 80.3 cM; [40]). Three other genes of interest are a thioredoxin, 1,4-benzoquinone reductase, and a protein kinase (markers *Swes578* - 51.6 cM, *CA651264* - 55.9 cM -*,* and *wPt-5011 -* 59.1 cM, respectively); these are positioned under the individual QTL *QPm.osu-1B*[41]. There are numerous markers that correspond to *NBS-LRR* genes positioned under individual QTL or MQTL (Additional file 8). These have particular importance as candidate genes. Indeed, some of these are similar to cloned *R* genes: the marker *wPt-1862* that is mapped under MQTL1 on chromosome 1A (45.1 cM) is very near to the *Pm3g* allele and has 44% identity at the amino-acid level with the *Pm3* gene. Three DArT markers, *wPt-1912*, *wPt-4107*, and *wPt-1560*, are mapped within the region of MQTL3 on chromosome 1B (15 cM, 16.1 cM, and 16.5 cM, respectively); the first two of these putatively correspond to NBS-LRR proteins, and the third to a protein kinase (Table 3). The marker *wPt-1912* in particular shows 69% identity at the amino-acid level with the *R* gene *Lr21* of *T. aestivum*. There are four other DArT markers that putatively code for NBS-LRR proteins within the region of MQTL13 on chromosome 4A (Table 3). One of these, *wPt-3729* (177.6 cM), shows 83% identity with the rust resistance *Rp1-like* gene of *T. aestivum*. Many genes of interest are associated with individual QTL on chromosome 7A. The markers *wPt-4487b* (36.2 cM) and *wPt-7491b* (43.1 cM) (LRR protein kinases), *wPt-6966* (36.6 cM), *wPt-3648* (41 cM), and *wPt-3434* (43.6 cM) (NBS-LRR proteins), and *wPt-1441* (37.9 cM) (acyl-protein synthetase) are positioned under QTL-7A on the short arm. The marker *wPt-6966*, in particular, shows 49% identity at the amino-acid level to the *B. distachyon* gene *Lr21*, for resistance to leaf rust. Examples like these make the *NBS-LRR* genes good candidates for the *R* genes and for the MQTL, and suitable markers to follow resistant phenotypes in a breeding program.

**Table 3:** Candidate genes mapped in the MQTL regions of the wheat genome

**Chr**	**MQTL**	**Locus name**	**Position**	**Putative function**
1A	MQTL1	wPt-1862	45.1	NBS-LRR
		Xcdo1160		ketol-acid reductoisomerase
1B	MQTL3	wPt-1912	15	NBS-LRR
		wPt-4107	16.1	NBS-LRR
		wPt-1560	16.5	LRR Protein kinase domain
		wPt-6592	18.6	Cation transporter/ATPase, N-terminus
2A	MQTL4	wPt-5865	69.4	Leucine-rich repeats (LRRs), ribonuclease inhibitor (RI)-like subfamily
2B	MQTL6	Xbcd265c	83	Tubulin alpha-3 chain
	MQTL8	BJ253815	124.8	Metallothionein
		Xcdo244	129.9	NBS-LRR
3A	MQTL10	Xbcd22	27.2	Glycosyl transferase family 8
		wPt-9049	42.4	PHD zinc finger protein-like
		wPt-2698	44.7	PHD zinc finger protein-like
		BJ213673c	45	CTD-phosphatase-like protein
		wPt-2938	48.4	WRKY transcription factor 30
		Xbcd828	50.1	H^+^ −transporting ATP synthase beta chain
		wPt-0714	52.1	HEAT repeat family protein
		Xmag620b	52.4	Zinc finger, C2H2 type
		TC74823a	64.8	SGNH_hydrolase
4A	MQTL12	Xbcd130	141.2	GDSL esterase/lipase
		Xbcd135b	143.8	Nucleolar protein,Nop52
		wPt-4596	147.6	BTB/POZ domain
	MQTL13	wPt-4487a	173.7	NBS-LRR
		wPt-4620a	176.7	ATP binding protein, putative
		wPt-0833a	176.8	NBS-LRR
		wPt-0992a	176.9	NBS2-RDG2A
		Xmag1140	177.1	Probable carboxylesterase 2-like
		Xmag3733	177.1	Probable carboxylesterase 2-like
		wPt-3859	177.2	Choline monooxygenase
		wPt-3729	177.6	NBS-LRR
5B	MQTL17	wPt-3569b	109.1	Serine threonine kinase
6A	MQTL20	wPt-8373	128.7	Cation efflux family protein-like
		wPt-7655	130.4	1,3-beta-glucan synthase component

*Chr* chromosome, *NBS* nucleotide binding site, *LRR* leucine-rich repeat.

As well as the information on putative functions, candidate genes were also searched for on the basis of the expression data available in the PLEXdb database. The available nucleotide sequences of markers positioned on the integrated map developed in the present study were searched for the corresponding probe-set on the wheat 61 K chip (http://www.plexdb.org/modules/tools/plexdb_blast.php), and the expression of each probe-set was then evaluated in two experiments in which wheat plants were subjected to infection with powdery mildew (TA34, TA39; Additional file 4). Some probe-sets were up-regulated in response to powdery mildew infection, and in particular those that correspond to the markers: *Xmag974* (155.9 cM), close to MQTL12 on chromosome 4; *wPt-5766* (24 cM), under *QPm.inra-5D*; *TC85303a* (73.1 cM); and *CA677684*, under *QPm.caas.6B*; *TC77994* (100.1 cM), very close to MQTL21 on chromosome 7A; and *wPt-4902* (160.4 cM), under *QPm_RF* on chromosome 7B.

The expression of some other probe-sets was down-regulated by powdery mildew infection, as for markers: *Xbcd1355* (87.9 cM), under MQTL15 on chromosome 5A; and *TC70722* (158.9 cM), under QPm_RF on chromosome 7B. The probe-sets that correspond to markers *TC85037* (76.4 cM) and *TC65966* (78.2 cM) under QPm.caas.6B were up-regulated and down-regulated in the two experiments taken into consideration. The probe-sets that correspond to five markers also showed differential expression between the resistant and susceptible genotypes studied in the framework of experiment TA39 (*TC85303a* - 73.1 cM on 6B, *TC85037 -*76.4 cM on 6B, *CA677684b* - 98.2 cM on 6B, *TC77994* -100.1 cM on 7A, and *TC70722* - 158.9 cM on 7B). More details are reported in Additional file 4.

Some chromosomes were characterized by the presence of resistance QTL and MQTL, but not of *Pm* genes, and therefore these appeared to bring only loci for quantitative resistance to powdery mildew, even if single genes responsible for qualitative resistance to other diseases have been reported previously, such as *Lr28* and *Lr30*, and *Sr7*, and *Stb7*, for resistance to leaf rust, stem rust and septoria, respectively, on chromosome 4A [42].

### Collinearity analysis

The availability of markers with a known sequence offers an opportunity to expand the collinearity analysis across grass genomes, moving from the wheat integrated map. The sequences of markers that correspond to expressed genes were used as queries in BLASTX searches, to identify segments of collinearity with the *Brachypodium* and rice genomes (http://www.phytozome.net/). We report the rice sequences with the ID reported by Phytozome, and we also add in round brackets the new Os ID, when available, according to RAP-DB (http://rapdb.dna.affrc.go.jp/) to make it easy to find the right gene position. Additional file 4 includes the results of the collinearity analysis for all of the DArT markers and the EST-SSRs positioned under individual QTL or MQTL, and for which there was a significant match on the *Brachypodium* and rice genomes. Syntenic regions were identified on durum-wheat chromosomes 2A, 4A, 5A, 6A, 7A, 1B, 2B, 3B, 5B, 6B, and 7B. Overall, many of the wheat genomic regions identified showed correspondence only with the *Brachypodium* genome; in other cases, correspondence was maintained also with the rice genome.

On chromosome 3A, there was correspondence across nine markers mapped on the wheat integrated map (*Xbcd22* - 27.2 cM, *BJ213673 - 45 cM*, *wPt-2938 - 48.4 cM*, *Xbcd828 - 50.1 cM*, *TC74823 - 64.8 cM*, *wPt-2202* - 72.7 cM, *wPt-6422* - 76.7 cM, *wPt-4077 -* 82.5 cM, and *Xbcd372* - 100.6 cM) and regions on chromosomes 2 and 1 of *Brachypodium* and rice, respectively (Additional file 4). This region on the map comprised a DArT marker that corresponds to a *NBS-LRR* gene (*wPt-4077*). The region of chromosome 1 of rice comprised between the loci Os01g47540 (Os01g0665300) and Os01g67800 contains 1,430 genes with descriptions. Expression data were searched for the genes included in this region of rice chromosome 1, to identify genes that might be involved in the rice reaction to powdery mildew. In particular, the genes where there was a 4-fold change in expression during non-host interactions with *Blumeria graminis* f. sp. *hordei* were selected from the plant expression database (experiment OS92 - PLEXdb; http://www.plexdb.org/modules/PD_browse/experiment_browser.php?experiment=OS92). A total of 15 genes with a 4-fold change were identified (Table 4), which included a putative LRK1 protein, a MDR-like ABC transporter, a NPK1-related protein kinase, a putative RING-H2 zinc finger protein, a putative peroxidase, and an S-adenosylmethionine-dependent methyltransferase. Most of the selected sequences belong to gene families known to have key roles in plant responses to pathogen attack.

**Table 4:** List of candidate genes in rice collinear regions with a 4-fold change in transcript levels in response to powdery mildew

**Chr**	**Marker interval wheat**	**Locus interval rice**	**Rice locus**	**BLASTX best hit**	**Putative function**	**E value**
3A	Xbcd22-Xbcd372	Os01g47540 (Os01g0665300)-Os01g67800	Os01g48610 (Os01g0677900)	NP_001043854	unknown	0.00E + 00
			Os01g50100 (Os01g0695800)	CAD59587	MDR-like ABC transporter	0.00E + 00
			Os01g50420 (Os01g0699600)	ACH99698	NPKL4	0.00E + 00
			Os01g51670 (Os01g0714600)	NP_001044058	unknown	1.00E-98
			Os01g53920 (Os01g0742400)	BAD87898	putative LRK1 protein	0.00E + 00
			Os01g55560 (Os01g0760900)	Q5JMF2	Abl interactor-like protein 5	0.00E + 00
			Os01g55974 (Os01g0765000)	BAD87146	deoxycytidylate deaminase-like	7.00E-164
			Os01g56560 (Os01g0772400)	EAY75988	putative coenzyme Q binding site	2.00E-133
			Os01g57730 (Os01g0787000)	BAB90103	putative peroxidase	0.00E + 00
			Os01g58170	BAD53004	S-adenosylmethionine-dependent methyltransferase	7.00E-35
			Os01g60730 (Os01g0822800)	BAB86495	putative RING-H2 zinc finger protein	2.00E-60
			Os01g62430 (Os01g0841700)	Q0JHU5	Elicitor-responsive protein 1	5.00E-105
			Os01g63380 (Os01g0852650)	EAZ14299	MULE transposase domain	0.00E + 00
			Os01g63920	BAD82052	unknown	9.00E-74
2B	wPt-2120-Xcdo244	Os04g22100-Os04g59494 (Os04g0691500)	Os04g30670	ADB85432	putative retrotransposon protein	1.00E-05
			Os04g34540 (Os04g0422700)	EAY94121	hypothetical protein	5.00E-30
			Os04g27020	NP_001052496	Cytochrome P450 family protein	0.00E + 00
			Os04g28140	CAE02105	unknown	4.00E-93
			Os04g47400 (Os04g0561900)	NP_001053555	unknown	0.00E + 00
			Os04g47650 (Os04g0564100)	EAZ41424	Peptidase S9A	3.00E-05
			Os04g38930 (Os04g0463300)	CAE76009	PPR repeat family	0.00E + 00
			Os04g39350 (Os04g0469000)	NP_001053041	unknown	5.00E-46
			Os04g53998 (Os04g0632100)	NP_001053986	S-locus receptor-like kinase RLK13	0.00E + 00
			Os04g57570	AAO37957	putative gag-pol polyprotein	0.00E + 00
4B	Xbcd110-wPt-9223	Os03g04410 (Os03g0136900)- Os03g16310	Os03g01740 (Os03g0107700)	Q10SY2	Cyclin-dependent protein kinase inhibitor EL2	3.00E-54
			Os03g03034 (Os03g0122300)	ABF93706	2OG-Fe oxygenase family protein	0.00E + 00
			Os03g07880 (Os03g0174900)	ABF94255	CCAAT-Binding transcription Factor	7.00E-161
			Os03g10100 (Os03g0197200)	EEE58504	Sugar transporter family protein	0.00E + 00
			Os03g11690 (Os03g0216300)	EEC74765	PPR repeat family	0.00E + 00
			Os03g10600 (Os03g0203100)	EEE58531	Urb2/Npa2 pre-60S ribosomal particles family	0.00E + 00
			Os03g15320 (Os03g0258900)	ABF95066	glyoxal oxidase	0.00E + 00
5B	-	Os09g01690 (Os09g0104300)-Os09g38790 (Os09g0560900)	Os09g10550	NP_001175748	Ulp1 protease family	4.00E-143
			Os09g11480 (Os09g0287000)	BAD29670	ethylene responsive AP2/ERF domain protein	1.00E-73
			Os09g21620 (Os09g0384300)	BAD25962	F-box-like protein	0.00E + 00
			Os09g23610 (Os09g0400900)	BAD28514	unknown	3.00E-30
			Os09g25520	BAD33414	unknown	0.00E + 00
			Os09g29710 (Os09g0472900)	NP_001063448	Blight-associated protein p12 precursor	1.00E-70
			Os09g31019 (Os09g0483200)	NP_001063507	Ribosomal L40e family	3.00E-82
			Os09g32360 (Os09g0499400)	BAF25485	unknown	0.00E + 00
			Os09g39080 (Os09g0564200)	EAZ45686	unknown	3.00E-39

*Chr* chromosome.

The same approach was used for a region on chromosome 2B comprised between the markers *wPt-2120* (74.4 cM) and *Xcdo244* (129.9 cM). There are corresponding regions on chromosome 5 (Bd5g03654-Bd5g27500) of *Brachypodium* and on chromosome 4 of rice [Os04g22100-Os04g59494(Os04g0691500)]. The rice genomic region contains 3,413 predicted genes, and based on expression data, 10 genes were identified with 4-fold changes, which included a pentatricopeptide repeat protein, a heavy-metal transport/ detoxification protein, a receptor-like protein kinase, and two retrotransposon proteins.

Three markers under MQTL14 on chromosome 4B, *Xbcd110* (107.4 cM), *wPt-7062* (109.6 cM), and *wPt-9223* (112.1 cM), identified collinear regions on chromosome 1 of *Brachypodium* (Bradi1g68460-Bradi1g75960) and chromosome 3 of rice [Os03g04410 (Os03g0136900)-Os03g16310]. Also in this case, seven genes that were in this region were differentially expressed in response to powdery mildew in rice, including a cyclin-dependent protein kinase inhibitor, an Fe oxygenase, a CCAAT-binding transcription factor, a sugar transporter, a glyoxal oxidase, and a pentatricopeptide repeat protein.

When possible, information on the physical mapping of genes for resistance to powdery mildew was used for the collinearity search. Blanco et al. [43] assigned the *Pm36* gene to chromosome bin 5BL6-0.29-0.76. A list of wheat ESTs was selected that were previously mapped to the same chromosomal region [44,45]; these were used to search the corresponding loci in rice (Additional file 9), and strong correspondence with chromosome 9 was identified. All of the predicted genes contained within the interval Os09g01690 (Os09g0104300)-Os09g38790 (Os09g0560900) were inspected for expression data, and there were nine genes with 4-fold changes, including a protein belonging to the Ulp1 protease family, an ethylene-responsive protein containing an AP2/ERF domain, an F-box-like protein, blight-associated protein p12, and a ribosomal protein. Furthermore, in a very recent study, six ESTs that derive from a wheat ‘totipotent’ cDNA library (*AJ609811*, *AJ610871*, *AJ611689*, *AJ614358*, *AJ716441*, and *FM208374*) were differentially expressed in two durum-wheat near-isogenic lines that differ in their resistance to powdery mildew, and some of them (*AJ610871*, *AJ611689*, *AJ716441*, and *FM208374*) were physically mapped to the centromeric bin region of chromosome 5BL, where the *Pm36* gene was localized previously [46]. Based on similarity searches, these sequences appear to correspond to the *EARLY RESPONSIVE TO DEHYDRATION* 15 (*ERD15*) transcription factor (*AJ611689*), an ATPase (*AJ716441*), and the heat-shock protein HSP90C (*FM208374*).

Following the approaches described above, a list of candidate genes was obtained, with each gene showing three fundamental features: (i) a map position coincident with a gene/ QTL/ MQTL of resistance to powdery mildew; (ii) a putative function related to disease response, and (iii) modulation of the expression levels by the pathogen. In particular, there were proteins belonging to the receptor-like kinase (RLK) family, genes involved in cell-wall modifications, NBS-LRR proteins, genes belonging to the ‘secretion and transport’ category, WRKY transcription factors, ABC transporters, peroxidases, and proteins involved in lipid and amino-acid metabolism.

### Homoelogous relationship between MQTL

Many markers that revealed multiple loci were positioned in the integrated map elaborated in the present study. The detailed analysis of homoelogous and nonhomoeologous positions for each marker enables us to propose some hypotheses relating to the evolution of loci for resistance to powdery mildew in wheat.

The markers *Xcfd15* (55.2 cM), *Xwmc818a* (57.4 cM), *XksuG9* (68.3 cM), and *Xgwm666* (72.8 cM), are positioned in the region of MQTL2 on chromosome 1A, and they show correspondence with homoelogous loci on chromosome 1B, under MQTL3. Furthermore, the markers *XksuG9* (45.1 cM), *Xgwm608* (55.2 cM), and *Xbarc119* (48.6 cM) reveal a third locus on chromosome 1D, under the individual QTL QPm.inra.1D.1. Altogether, these data suggest a homoelogous relationship across MQTL2, MQTL3, and the QTL QPm.inra.1D.1 in group 1. The absence of homoelogous loci between the regions of MQTL1 and MQTL3 supports the results of the meta-analysis and that MQTL1 is separated from MQTL2. A group of markers (*Xgwm165a* - 13.9 cM; *b* - 105.2 cM, *Xwmc89a* - 15.5 cM; *b* - 106.6 cM, *Xwmc491a −*15.8 cM; *b* - 107.2 cM, *Xwmc617b* - 28.5 cM; *a* - 103.8 cM, and *Xgwm192a* - 38.5 cM; *b −*107 cM - between 4A and 4B; and *Xwmc617a; c* - 0.0 cM, *Xgwm165b*; *c* - 38 cM, *Xgwm608d* - 105.5 cM; *e* - 31 cM, *Xwmc206b* - 106.6 cM; *e* - 54 cM, *Xcfd39a* - 103.1 cM; *b* - 57 cM, and *Xwmc89b*; *c* - 27 cM - between 4B and 4D) identified loci in homoelogous positions on chromosomes 4A, 4B and 4D, which suggests that there might be a homoelogous relationship across MQTL11, MQTL14, and the individual QTL QPm.caas.4D on chromosomes 4A, 4B, and 4D, respectively. The same relationship was found for MQTL20 and the individual QTL PMm on chromosomes 6A and 6B, respectively, based on markers *Xabc175* (*b* - 81.9 cM; *a* - 98.1 cM), *Xwmc179* (*b* - 64.7 cM; *c* - 95.5 cM), *wPt-3191* (*a* - 114.6 cM; *b* - 98.2 cM), *Xwm417* (*a* - 121.7 cM; *b* - 111.9 cM), *Xcdo1091* (*a* - 125.4 cM; *b* - 104.3 cM), and *Xpsr546* (*a* - 138.2 cM; *b* - 108 cM), and between MQTL22 and MQTL23 on chromosomes 7A and 7B, respectively, based on markers *Xgwm746* (*a* - 172.7 cM; *b* - 110.1 cM), *wPt-6869* (*a* - 200.7 cM; *b* - 148.9 cM), *wPt-3439* (*a* - 202.5 cM; *b* - 151.2 cM), *Xcfa2040* (*b* - 202.7 cM; *a* - 151.5 cM), *Xgwm344* (*b* - 208.8 cM and *c* 153 cM; *a* - 234.6 cM and *d* - 182.7 cM), and *Xgwm1061* (*a* - 210.7 cM; *b* - 162.1 cM). In all of these cases, resistance determinants might have originated before diversification of homoelogous genomes.

The case of MQTL6 was different, which was mapped on chromosome 2B. No QTL or *Pm* genes were identified in the homoelogous region on chromosome 2A based on the markers *Xgwm630* (*a* - 62.1 cM; *b* - 81.3 cM), *XksuF37* (*a* - 61.3 cM; *b* - 82.5 cM), *Xwmc474*,(*a* - 56.1 cM; *b* - 82.7 cM), and *wPt-2120* (*a* - 57.4 cM; *b* - 73.3 cM). In this case, there might be a homoelogous QTL, although it has not been mapped yet, or a resistance source might have evolved after diversification of the homoelogous genomes, or even before, but followed by the loss of the resistance determinant on chromosome 2A.

**Figure 1:**
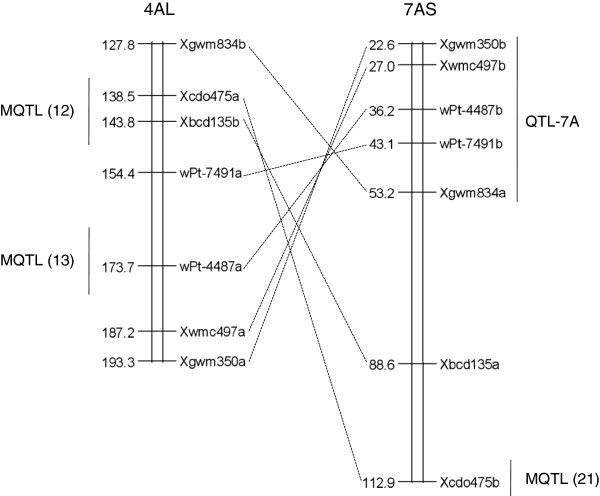
Partial genetic map of the regions on chromosomes 4AL and 7AS corresponding to the translocation 7B:4A.

Groups of multi-locus markers that reveal loci mapped on nonhomoeologous positions were considered to be involved in putative translocations, as described by Marone et al. [22]. In particular, the translocations that involve chromosome arms 4AL, 5AL, and 7BS have been firmly established [22,47-56]. At the diploid level, an exchange of the terminal segments of chromosomes 4AL and 5AL took place, followed in tetraploid wheat by the exchange of the distal portion of chromosome 5A segment on chromosome 4AL with a terminal segment from chromosome 7BS. In the present study, a group of seven markers reported in Figure 1 identified the loci on the short arm of chromosome 7AS and on the long arm of chromosome 4A, instead of chromosome 7B; this allowed us to identify the translocation event that took place between homoelogous groups 4 and 7 on the integrated map developed in the present study. As previously shown [22], the order of the markers was inverted in the two regions. More interestingly, individual QTL and MQTL were positioned in the translocated region. In particular, a correspondence can be seen between the region of MQTL12 on chromosome 4A and MQTL21 on chromosome 7A. Furthermore, the position of the two loci revealed by the marker *wPt-4487* (*a* - 173.7 cM; *b* - 36.2 cM) suggests a correspondence between MQTL13 on 4AL and the individual QTL-7A on 7AS. These results strongly suggest that the loci for resistance to powdery mildew evolved during very ancient times, before this translocation event took place in wheat. Local rearrangements might then have driven the evolution of new resistance specificities in the described loci.

Another translocation event is underlined by a group of markers that reveal loci on chromosomes 5B and 7B (*Xbarc4a* - 100.5 cM - and *b* - 80 cM, *wPt-9814a* - 102.9 cM - and *b* - 81.8 cM, *wPt-5737a* - 103.1 cM - and *b* - 81.7 cM, *Xgwm68a* - 107.4 cM - and *b* - 72.6 cM, *wPt-1457a* - 108.1 cM - and *b* - 78.4 cM, and *Xbarc176a* - 109.5 cM - and *b* - 89.3 cM). All of these markers are within the region of MQTL17 on chromosome 5B, and correspond to the individual QTL QPmV.inra.7B on chromosome 7B.

### Analysis of sources of resistance to powdery mildew in wheat

The genetic background of resistant varieties, as the parents of the populations used to map genes and QTL responsible for powdery mildew resistance, was analyzed by searching for information in the Wheat Pedigree and Identified Alleles of Genes database (http://genbank.vurv.cz/wheat/pedigree/). A detailed investigation of the pedigree data of resistant varieties that is reported in Additional file 10 allowed us to hypothesize the origin of resistance and its occurrence in other cultivars characterized by common ancestors. Twenty resistance genes mapped in the present study are already known from the literature to be transferred from diploid and tetraploid relatives of wheat, including *T. turgidum* var. *dicoccoides* (*MlIW170*, *Pm42*, *MlAB10*, *MlZec1*, *Pm41*, *Pm16*, *Ml3D232*, *PmAS846*, *Pm36*, *PmG3M*, *PmG16*, and *MlIW72*), *T. timopheevii* (*Pm27*, *Pm37*, *PmNCAG11*, and *MlAG12*), *T. carthlicum* (Nevski in Kom.) (*Pm6* and *Pm33*), *T. monococcum* (*Pm4d*, *Pm2026*, *NCA6Pm*, *Mlm2033*, and *PmNCA4*), *T. boeoticum* (Boiss) (*PmTb7A.1*, *PmTb7A.2*, and *Mlm80*), *T. urartu* (Tum.) (*PmU*) and several *Aegilops* spp. (*PmY212*, *Pm35*, *PmY201*, *Pm34*, and *Pm12*) or from related genera, such as *Elytrigia* (*Pm40*), *Secale* (*PmHNK54* and *PmTm4*), and *Thinopyrum intermedium* (Host) (*Pm43*) (Additional file 10). In addition, there were MQTL that grouped single QTL that were contributed by the same wild ancestor. For example, MQTL2 combined the two QTL mapped in a population that derives from a cross between *T. militinae* (Zhuk.) and a wheat cultivar [57,58], and one QTL where the resistance resulted from a variety that had *T. timopheevii* in its genetic background. As *T. militinae* derives from *T. timopheevii*, the resistance source might be the same. MQTL3, 13, 16, and 17 were also characterized by *T. timopheevii*-derived resistance. In contrast, MQTL that derive specifically from *T. aestivum* were also identified, such as MQTL1 (Additional file 10). This demonstrates that it is still possible to exploit the cultivated wheat to search for new resistance genes.

The coincidence of single QTL that co-map with resistance genes that derive from the same species was verified, as for chromosome 1D on which *Pm24* was located under the QTL QPm.inra [59], where both derive from *T. aestivum*. An analogous case was on chromosome 6B, where *Pm27* and the QTL PmM [60] co-map, and both derive from *T. timopheevii*.

The convergence in the same region of QTL and genes that derive from different species was the most represented case along the chromosomes of the wheat genome, which leads to the hypothesis that different sources of resistance might have contributed to these regions. Chromosome 1A was characterized by the presence of introgression from *T. militinae*, which started from the marker *Xpsp2999* (47.2 cM), as proposed by Jakobson et al. [58], where MQTL1 and MQTL2 were located, also including *Pm3g*, *Pm3a*, *Mlar*, and *Pm3e*, where their resistance derives instead from *T. aestivum*. A gene-rich region was identified on chromosome 2A, corresponding to MQTL4. This region appears to have been subjected to multiple introgression events from wild species, as genes and QTL that derive from different sources were mapped (*T. aestivum*, *T. dicoccum*, *T. monococcum*, *T. timopheevii*, and *T. carthilicum*). There are other intriguing regions in which different sources of resistance contributed to the same chromosomal region on chromosomes 2B (*T. aestivum*, *T. carthlicum*, and *T. dicoccoides*), 5A (*T. aestivum*, *T. monococcum*, and *T. militinae*), 5B (*T. aestivum* and *T. dicoccoides*), and 6B (*T. aestivum*, *Ae. Speltoides, T. timopheevi,* and *T. dicoccoides*). In particular, the long arms of chromosomes 7A and 7B are characterized by a large number of genes/ QTL with different resistant sources, which are localized at small distances. Finally, the analysis of pedigree performed in the present study reveals that common wheat varieties recur in the genetic background of most of the resistant varieties used to map powdery mildew resistance genes/ QTL. In particular, the American cultivar ‘Mediterranean’, the Japanese landrace ‘Akakomugi’, the French cultivar ‘Chiddam d’automne’ and the Japanese cultivars ‘Norin10’ and ‘29’ recur in most of the pedigrees analyzed (Additional file 10).

## Conclusions

This QTL meta-analysis has allowed us to reduce the CIs of the resulting MQTL relative to those of the initial QTL by a coefficient of reduction of 3.2 (mean initial CI/ mean MQTL CI), thus facilitating the search for candidate genes and providing markers that are more closely associated with the corresponding MQTL.

Meta-analysis of QTL for powdery mildew resistance is an effective approach to identify precise consensus QTL, which allows the confusion that exists due to redundancy in the number of QTL in overlapping genomic regions to be overcome. The development of an integrated map that is very dense and rich in markers that correspond to expressed genes that have putative roles in plant responses to pathogens represents a useful resource for the analysis of multiple components of resistance to diseases. The 24 MQTL identified that show small genetic intervals represent an important tool that can be used for marker-assisted selection/ pyramiding in wheat-breeding programs, or for map-based cloning. The presence of clusters of genes in the regions involved in the control of this trait indicates that a well-coordinated response of many genes is fundamental to achieve pathogen resistance. The comparative genomics approach to identify the consistency of QTL for resistance against *B. graminis* across grass genomes reveals the conservation and evolutionary significance of some of these loci. The information generated in this study will be of great use for future studies aimed to improving the powdery mildew resistance in wheat.

## Methods

### QTL mapping for powdery mildew resistance

The Creso × Pedroso mapping population was composed of 123 RILs and was evaluated during the growing season of 2005–2006 in Córdoba, southern Spain, and in Foggia, southern Italy, two of the major durum-wheat-growing areas in the Mediterranean basin. These 123 RILs and the parental cultivars Creso and Pedroso were sown in single 1-m-long rows, with 25 cm between rows, and organized in a randomized block design with three replications. No artificial inoculations were performed, as heavy infections of powdery mildew usually occur in these areas. All of the recommended agronomic practices were followed according to local standards. The disease severity was estimated visually when the disease was maximally spread, as the percentage of leaf area covered by powdery mildew in adult plants, at the level of the whole plant canopy. The parents Creso and Pedroso were characterized by a nearly identical heading time (data not shown), and the small differences observed across the segregating populations did not produce relevant effects of growth stage on the evaluation of disease severity. Phenotypic data were used for QTL mapping, together with the Creso × Pedroso genetic map that was previously developed [22,25]. Genome-wide QTL searches were conducted using the MapQTL software package, version 5.0 [61], using both the simple interval mapping and the multiple QTL mapping functions. The LOD profiles from simple interval mapping were examined, and the marker closest to each LOD peak was selected as the cofactor to perform the multiple QTL mapping analysis. The LOD significance threshold levels of the respective traits and the corresponding map were calculated with the permutation test option provided in MapQTL, using 10,000 permutations. The LOD threshold was 3.0.

### Bibliographic collection of QTL/ gene mapping

Twenty-three previously published studies were identified that reported on QTL for resistance against powdery mildew in bread wheat and durum wheat. Out of these, 20 were based on 19 different segregating populations, and these provided sufficient information on mapping and QTL characteristics for map projection and the MQTL (Additional file 6). Moreover, the information on the mapping data of resistance genes was collected from 58 published reports. Out of these, the projection of 52 genes was performed according to the common markers between the integrated map and the single genetic maps. For each gene, we defined the mapping population and the source of resistance, when known (Additional file 7).

### Development of a consensus map

An integrated genetic map of the A, B, and D wheat genomes was constructed using a bread-wheat consensus map developed by Crossa et al. [23] as the reference map, on which two other consensus maps were projected: the Somers consensus map [24] and the most recent durum-wheat consensus map [22]. All of the calculations for the creation of the integrated map were performed with the Biomercator software, version 2.1 [14]. Maps of chromosomes with fewer than two common markers to the reference map were excluded before the creation of the consensus map. The marker order was finally verified according to the single genetic maps, and possible inversions were filtered out by discarding inconsistent loci. Genetic maps containing genes and QTL for resistance to powdery mildew were also integrated, to determine their positions on the consensus map. Moreover, the available wheat chromosomal regions containing DArT markers for which a function associated to plant pathogenesis was previously suggested [25] were collected [62-71]; these were integrated into the consensus map to facilitate the search for candidate genes.

### QTL projection and meta-analysis

Quantitative trait loci were projected onto the consensus map using the Biomercator software. The 95% CIs of the initial QTL on their original maps were calculated through the empirical formula proposed by Darvasi and Soller [72], and extended by Guo et al. [73] according to the population type. For backcross, F_2_, and double-haploid populations, the QTL CI was 530/ N × R^2^, where N is the population size, and R^2^ is the proportion of the phenotypic variance explained by the QTL. The formula CI = 163/ N × R^2^ was used when a RIL population was considered. This approach is important, to assess the CIs using the same method for each QTL and study, and to estimate these when they were not published. In some cases, the calculated CI for a single QTL did not correspond to that reported in the literature, probably due to the small population size used to map the QTL, the very low percentage of explained variability, the method used for the QTL detection, or a sparse genetic map, which thus resulted in a broader interval on the integrated map [74,75]. Co-localizing QTL that derive from the same experimental population used in different studies were considered if the fungal race or plant growth stage analyzed were not identical. This reduced the pre-selected QTL (148) to 96 (Additional file 6).

Meta-analysis was carried out on the QTL cluster on each chromosome separately, and the MQTL were obtained from the midpoint positions of the overlapping QTL. For n individual QTL, the Biomercator software tests the most likely assumption between 1, 2, 3, 4, and n QTL. The Akaike Information Criterion (AIC) was considered to select the best QTL model that indicated the number of MQTL. The model with the lowest AIC value was considered the best fit [75]. When the n-model was the most likely model, the meta-analysis was performed again, choosing a subset of the QTL.

### Analysis of collinearity

Collinearity was investigated for all of the markers positioned on the integrated map for which a gene sequence was available. The sequences were used as queries in a BLASTX search against the *Brachypodium distachion* and rice protein sequences available in the Phytozome database (http://www.phytozome.net). Information regarding the expression of the rice genes comprised in synthenic regions was retrieved from the Plant Expression Database (PLEXdb; http://www.plexdb.org/modules/PD_browse/experiment_browser.php?experiment=OS92).

## Competing interests

The authors declare no competing financial interests.

## Authors’ contributions

DM and MAR developed the integrated map and carried out the QTL meta-analysis. GL, AG and AMM carried out the collinearity study. DR developed the Creso × Pedroso segregating population. PDV and DR carried out the phenotyping of the Creso × Pedroso segregating population. RP and AB provided general guidance for the study. DM and AMM drafted the manuscript. AMM conceived and coordinated the study. All of the authors have read and approved this version of the manuscript.

## Supplementary Material

Additional file 1 Phenotypic variation among the parental lines and RILs from the durum-wheat Creso × Pedroso population for disease severity caused by powdery mildew. Click here for file

Additional file 2 Results of the QTL analysis for resistance to powdery mildew in the Creso × Pedroso population. Click here for file

Additional file 3: Figure S1 Integrated map in wheat of the QTL and the MQTL identified by meta-analysis, for powdery mildew resistance. Vertical lines on the right of chromosomes indicate the confidence intervals, horizontal lines indicate the peak marker positions, where the length represents the percentage of variability explained by the QTL. The MQTL are in bold, with the single QTL in gray. The names of the QTL grouped in the same MQTL are in the same color. *Pm* genes are blue, markers that correspond to NBS-LRR proteins are purple, markers that correspond to kinases are brown. Markers where there is correspondence with probe-sets differentially expressed following powdery mildew infection are circled in black. Click here for file

Additional file 4 **The integrated map (in Excel format) with the markers for which a sequence is available and a putative function is annotated in public databases.** The information given is for the functional markers positioned within the individual QTL and MQTL regarding the collinearity analysis and the correspondence with probe-sets of the wheat 61 K array. Click here for file

Additional file 5 Results of the similarity search against the database of wheat separate chromosome arms promoted by the International Wheat Genome Sequencing Consortium. Click here for file

Additional file 6 Literature sources used in the meta-analysis of the QTL for resistance to powdery mildew. Click here for file

Additional file 7 **List of the *Pm *genes projected onto the integrated map.** Click here for file

Additional file 8 Co-localization of the QTL and MQTL with disease-resistance-related genes. Click here for file

Additional file 9 Collinearity between the physically mapped wheat ESTs and the rice genome. Click here for file

Additional file 10 Summary of the pedigree and sources of resistance for the QTL and genes mapped onto the integrated map. Click here for file
