# Supplementary material for: Genetic basis of qualitative and quantitative resistance to powdery mildew in wheat: from consensus regions to candidate genes
Source: BMC Genomics. 2013 Aug 19;14:562. doi: 10.1186/1471-2164-14-562 (PMC3765315; doi:10.1186/1471-2164-14-562)
Supplement: Additional file 1 — Phenotypic variation among the parental lines and RILs from the durum-wheat Creso × Pedroso population for disease severity caused by powdery mildew. [file 1471-2164-14-562-S1.docx]

**Additional File 1.** Phenotypic variation among the parental lines and RILs from the durum-wheat Creso × Pedroso population for disease severity caused by powdery mildew.

|  | **Italy 06** | **Spain 06** | **Pooled** |
| --- | --- | --- | --- |
| Creso | 47.5 | 23.3 | 35.4 |
| Pedroso | 72.5 | 35.0 | 53.7 |
| RIL mean | 48.3 | 30.5 | 39.1 |
| RIL range | 0-80 | 10-70 | 0-80 |
| CV (%) | 28.8 | 18.4 | 27.1 |
| LSD_0.05_ | 22.5 | 10.6 | 17.1 |

CV, coefficient of variation; LDS_0.05_, least differences significant at a probability level of *P* <0.05.
