# Supplementary material for: Genetic basis of qualitative and quantitative resistance to powdery mildew in wheat: from consensus regions to candidate genes
Source: BMC Genomics. 2013 Aug 19;14:562. doi: 10.1186/1471-2164-14-562 (PMC3765315; doi:10.1186/1471-2164-14-562)
Supplement: Additional file 2 — Results of the QTL analysis for resistance to powdery mildew in the Creso × Pedroso population. [file 1471-2164-14-562-S2.docx]

| **QTL** | **Position (cM)** | **Peak marker** | **IC (cM)** | **Chr** | **Trait** | **LOD** | **R^2^ (%)** | **Add. eff.** |
| --- | --- | --- | --- | --- | --- | --- | --- | --- |
| CP 1 | 56.461 | MAG1200b | 10.5 | 6A-1 | Italy | 3.61 | 12.6 | 0.401376 |
| CP 2 | 3.943 | F103 | 12.5 | 3B-1 | Italy | 3.00 | 10.6 | -0.36911 |
| CP 3 | 73.074 | Xgwm219 | 9 | 6B-2 | Italy | 3.27 | 14.8 | 0.473661 |
| CP 3 | 57.168 | Xgwm889 | 7.2 | 6B-2 | Spain | 5 | 18.5 | 2.64136 |
| CP 4 | 109.874 | wPt-5270 | 9.9 | 6B-2 | Spain | 3.6 | 13.4 | 2.21857 |
| CP 5 | 21 | wPt-5513 | 10.8 | 2B-1 | Spain | 3.08 | 12.3 | 2.21232 |

**Additional File 2.** Results of the QTL analysis for resistance to powdery mildew in the Creso × Pedroso population.

QTL, quantitive trai locus; IC, interval of confidence; Chr, chromosome; LOD, logarithm of odds; Add. eff., additive effect.
