# Supplementary figures and images for: Genetic basis of qualitative and quantitative resistance to powdery mildew in wheat: from consensus regions to candidate genes
Source: BMC Genomics. 2013 Aug 19;14:562. doi: 10.1186/1471-2164-14-562 (PMC3765315; doi:10.1186/1471-2164-14-562)

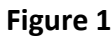

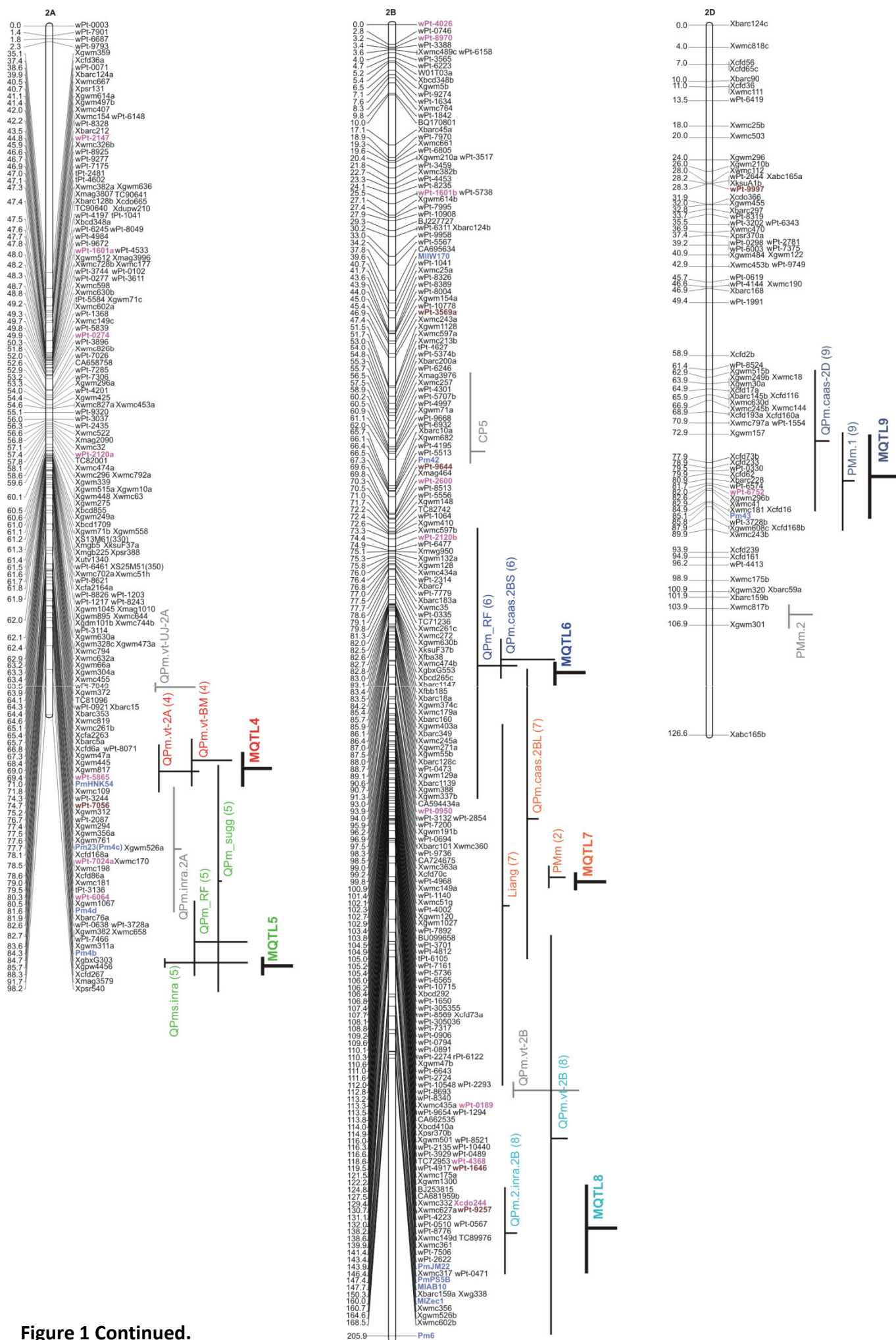

Figure 1 Continued.

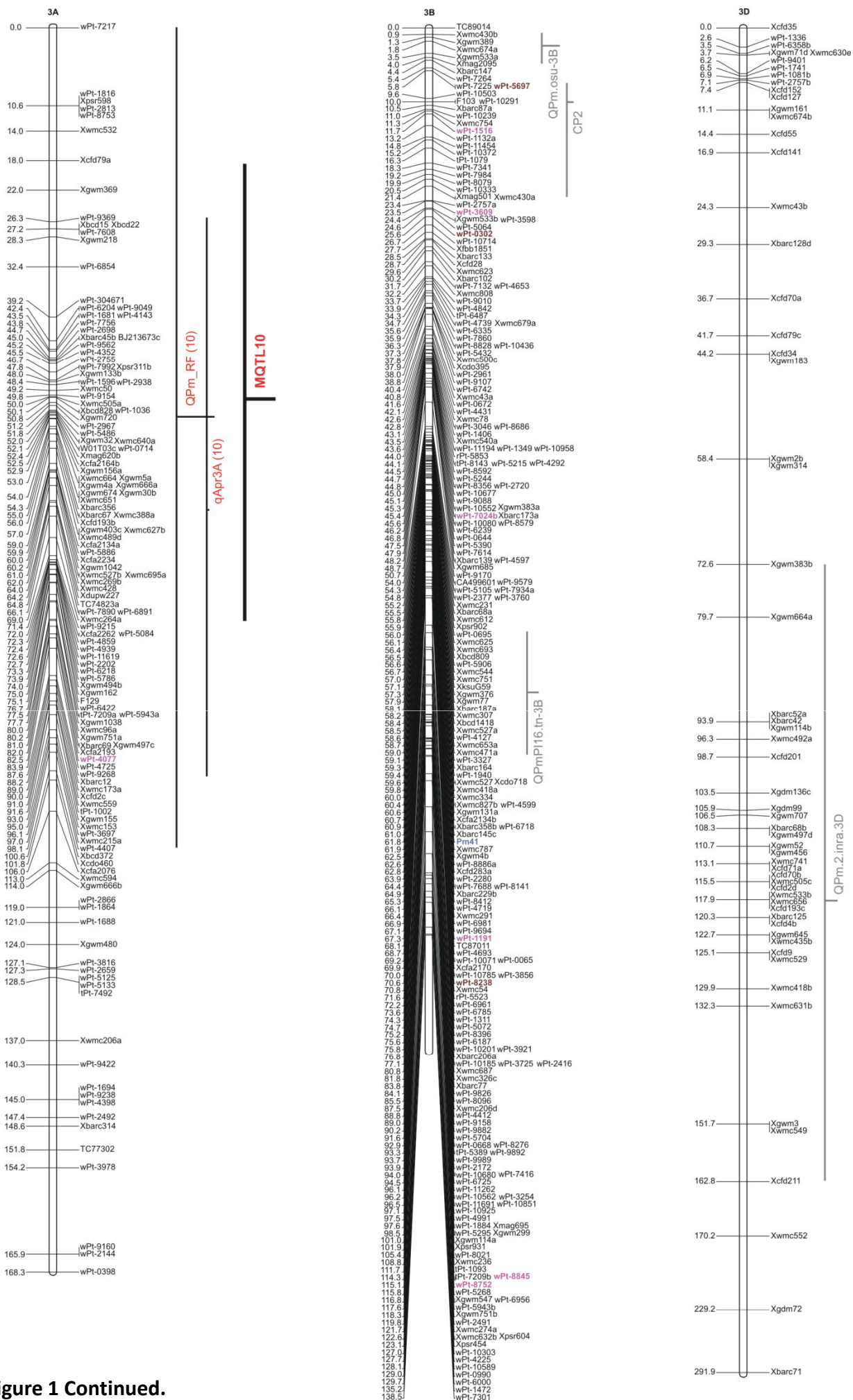

Figure 1 Continued.

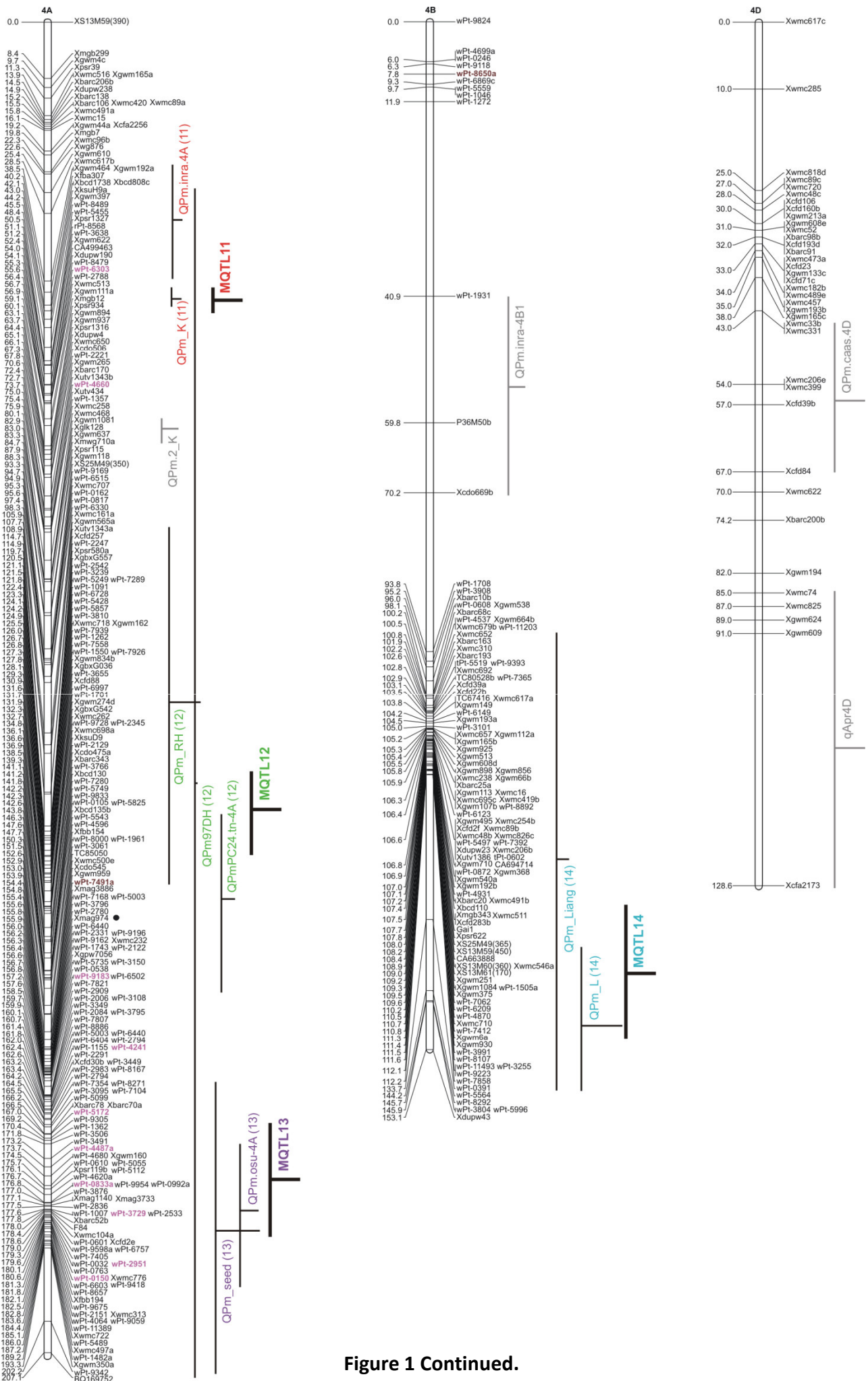

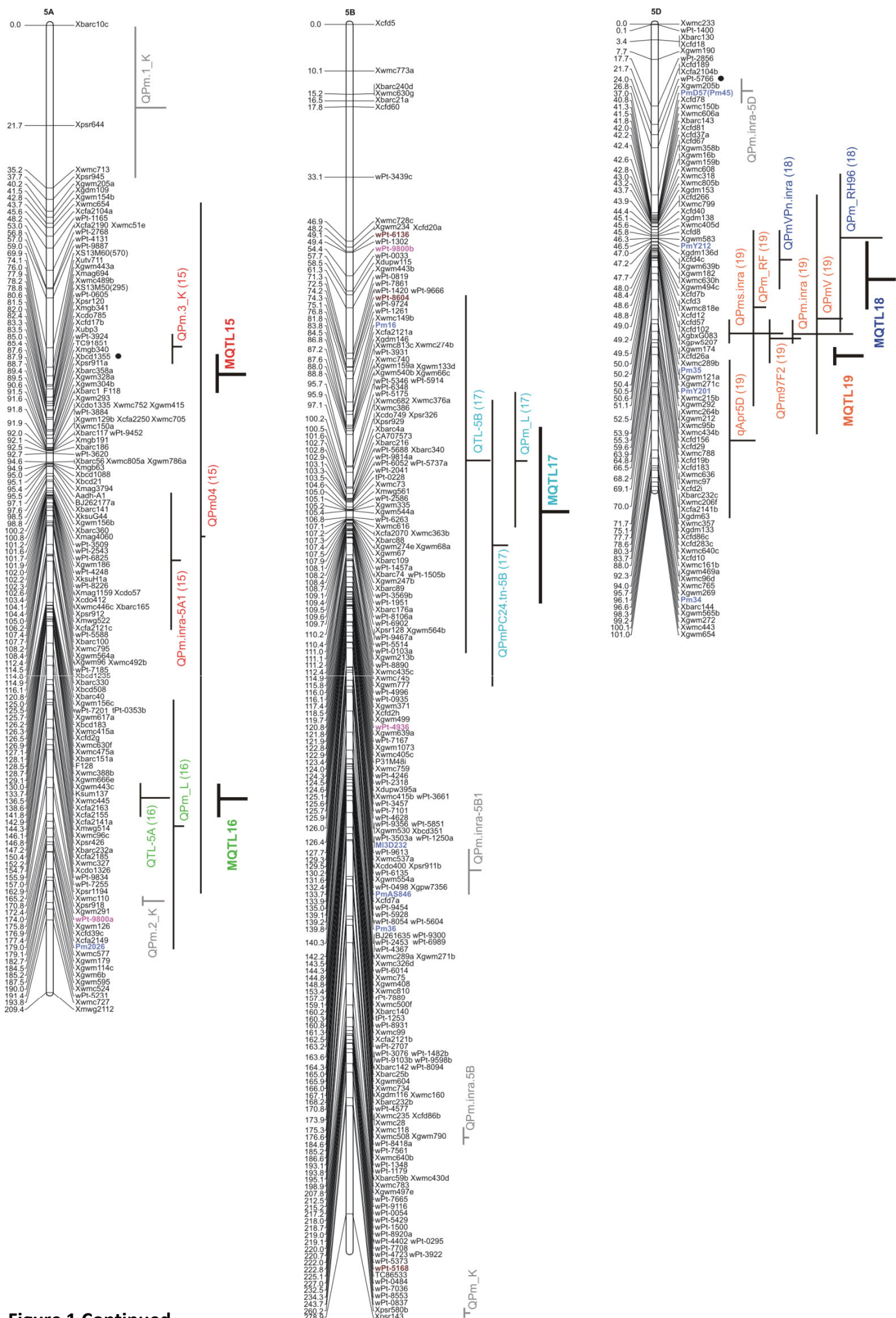

Figure 1 Continued.

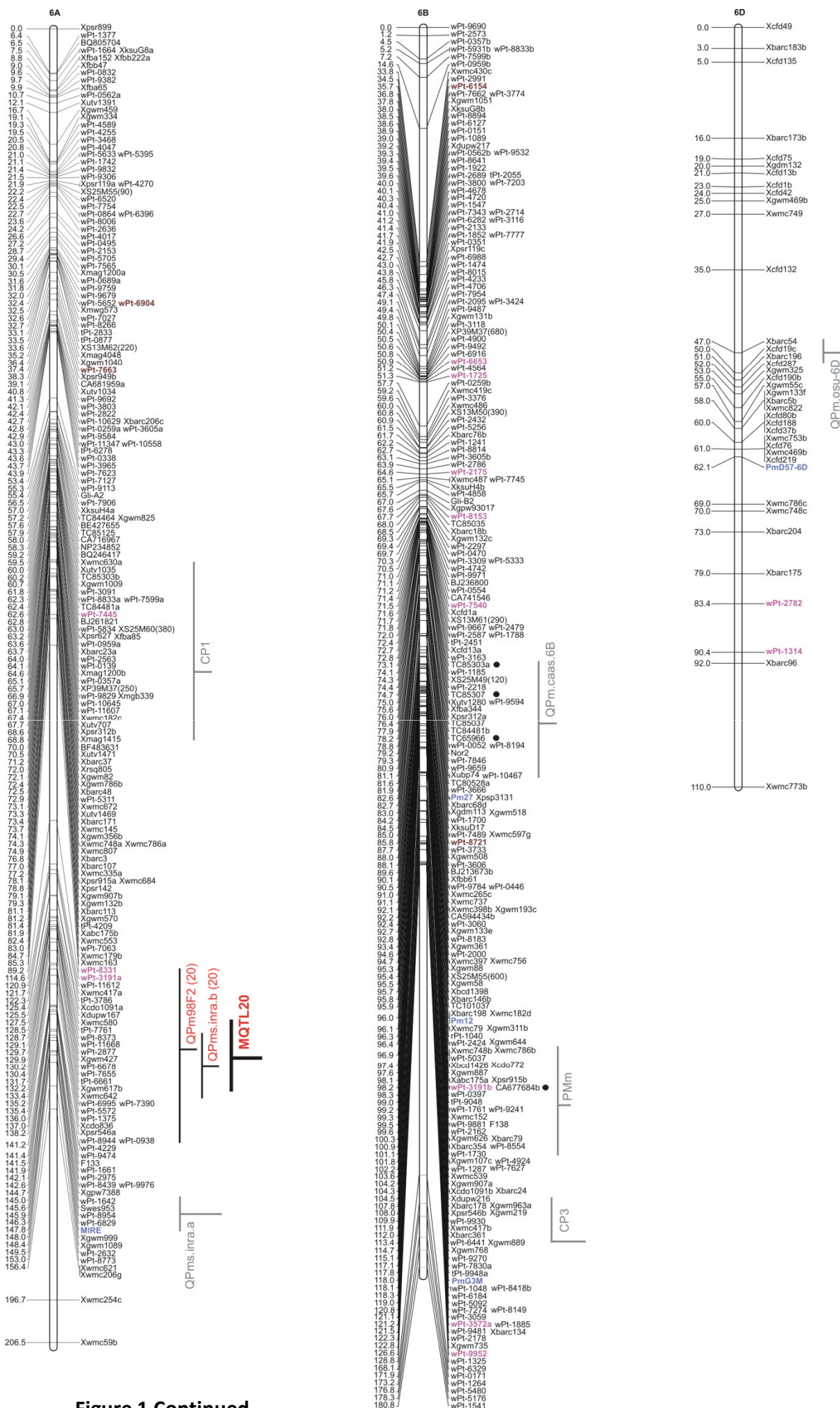

Figure 1 Continued.

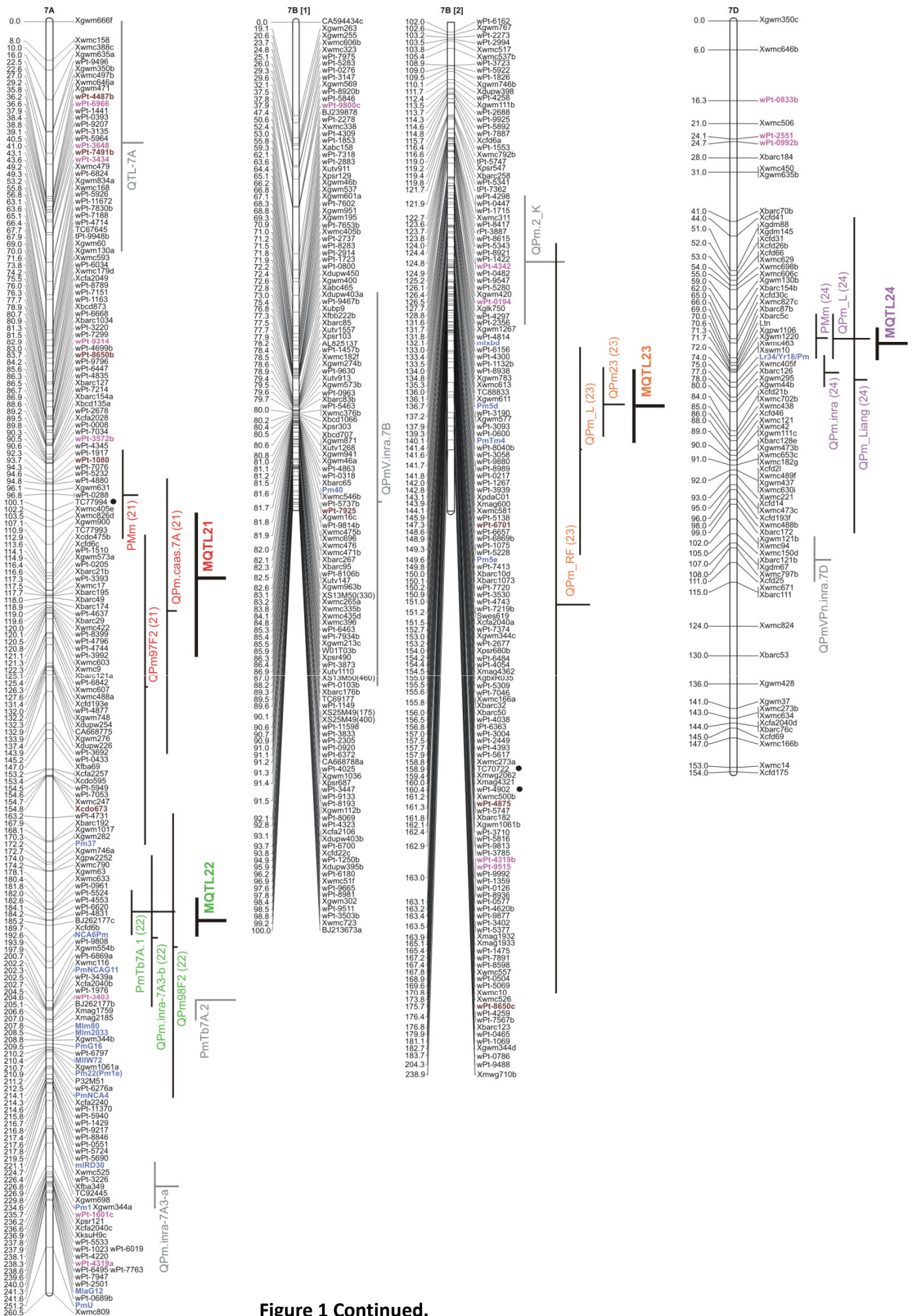

Figure 1 Continued.

Supplement: Additional file 3: Figure S1 — Integrated map in wheat of the QTL and the MQTL identified by meta-analysis, for powdery mildew resistance. Vertical lines on the right of chromosomes indicate the confidence intervals, horizontal lines indicate the peak marker positions, where the length represents the percentage of variability explained by the QTL. The MQTL are in bold, with the single QTL in gray. The names of the QTL grouped in the same MQTL are in the same color. Pm genes are blue, markers that correspond to NBS-LRR proteins are purple, markers that correspond to kinases are brown. Markers where there is correspondence with probe-sets differentially expressed following powdery mildew infection are circled in black. [file 1471-2164-14-562-S3.pdf]
