# Supplementary material for: Genetic basis of qualitative and quantitative resistance to powdery mildew in wheat: from consensus regions to candidate genes
Source: BMC Genomics. 2013 Aug 19;14:562. doi: 10.1186/1471-2164-14-562 (PMC3765315; doi:10.1186/1471-2164-14-562)
Supplement: Additional file 6 — Literature sources used in the meta-analysis of the QTL for resistance to powdery mildew. [file 1471-2164-14-562-S6.docx]

**Additional File 5.** Literature sources used in the meta-analysis of the QTL for resistance to powdery mildew.

| **Total No QTL** | **QTL used in meta-analysis** | **Mapping population** | **Population size** | **Population type** | **Stage of disease assessment** | **Type of inoculation** | **Total no of environments or locations** | **No of years** | **Analysis method** | **Reference** |
| --- | --- | --- | --- | --- | --- | --- | --- | --- | --- | --- |
|  |  |  |  |  |  |  |  |  |  |  |
| 10 | 8 | RE9001 x Courtot | 104 | RIL | adult plant | natural infection | 2 | 2 | CIM | [74] |
| 2 | 2 |  |  |  | vernalized seedling | artificial infection | 2 | 1 |  |  |
| 4 | 2 |  |  |  | vernalized seedling | natural infection | 2 | 1 |  |  |
|  |  |  |  |  |  |  |  |  |  |  |
| 3 | 1 | RE714 x Hardi | 44 | DH | adult plant | natural infection | 1 | 3 | SIM | [76] |
| 2 | 1 | RE714 x Hardi | 44 | DH | vernalized seedling | artificial infection | 1 | 3 |  |  |
| 4 | 3 | RE714 x Hardi | 140 | F2:3 | adult plant | natural infection | 1 | 2 |  |  |
|  |  |  |  |  |  |  |  |  |  |  |
| 5 | 5 | Jagger x 2174 | 96 | RIL | seedling stage | natural infection | 2 | 1 | SIM | [56] |
|  |  |  |  |  |  |  |  |  |  |  |
| 6 | 5 | Tahti x T. militinae | 130 | F2:3 | seedling stage and adult plant | natural infection | 1 | 2 | CIM | [72] |
|  |  |  |  |  |  |  |  |  |  |  |
| 18 | 8 | Forno x Oberkulmer | 204 | RIL | adult plant | natural/artificial infection | 5 | 2 | CIM | [77] |
|  |  |  |  |  |  |  |  |  |  |  |
| 4 | 4 | Bainong 64 × Jingshuang 16 | 181 | DH | adult plant | artificial infection | 2 | 2 | CIM | [55] |
|  |  |  |  |  |  |  |  |  |  |  |
| 3 | 3 | Lumai 21 x Jingshuang 16 | 200 | F3 | adult plant | artificial infection | 2 | 2 | CIM | [12] |
|  |  |  |  |  |  |  |  |  |  |  |
| 4 | 4 | Fukuho-komugi × Oligoculm | 107 | DH | adult plant | artificial infection | 2 | 2 | CIM | [78] |
|  |  |  |  |  |  |  |  |  |  |  |
| 7 | 6 | Avocet-YrA x Saar | 113 | RIL | adult plant | natural infection | 2 | 2 | SIM | [79] |
|  |  |  |  |  |  |  |  |  |  |  |
| 3 | 2 | Becker x Massey | 180 | F2:3 | adult plant | natural infection | 1 | 1 | SIM | [80] |
|  |  |  |  |  |  |  |  |  |  |  |
| 2 | 2 | Atlantis x Cortez | 91 | DH | adult plant | artificial infection | 6 | 4 | MIM | [81] |
|  |  |  |  |  |  |  |  |  |  |  |
| 4 | 4 | RE714 x Hardi | 160 | RIL | seedling stage | artificial infection | 1 | 1 | CIM | [82] |
|  |  |  |  |  |  |  |  |  |  |  |
| 7 | 6 | RE714 x Hardi | 160 | RIL | adult plant | natural infection | 1 | 3 | CIM | [83] |
|  |  |  |  |  |  |  |  |  |  |  |
| 7 | 5 | RE714 x Festin | 41 | DH | adult plant | natural infection | 3 | 3 | SIM | [84] |
|  |  |  |  |  |  |  |  |  |  |  |
| 2 | 2 | RE714 x Hardi | 44 | DH | adult plant | natural infection | 3 | 2 |  |  |
|  |  |  |  |  |  |  |  |  |  |  |
| 3 | 2 | Becker x Massey | 180 | F2:3 | adult plant | natural infection | 1 | 1 | CIM | [85] |
|  |  |  |  |  |  |  |  |  |  |  |
| 3 | 1 | USG 3209 x Jaypee | 293 | RIL | adult plant | natural infection | 1 | 2 |  |  |
|  |  |  |  |  |  |  |  |  |  |  |
| 27 | 3 | W7984 × Opata85 | 114 | RIL | NA | artificial infection | NA | NA | CIM | [86] |
|  |  |  |  |  |  |  |  |  |  |  |
| 3 | 3 | Huapei 3 x Yumai 57 | 168 | DH | adult plant | NA | 4 | 2 | CIM | [11] |
|  |  |  |  |  |  |  |  |  |  |  |
| 2 | 2 | Tb5088 x Tm14087 | 148 | RIL | adult plant | artificial infection | 1 | 1 | CIM | [87] |
|  |  |  |  |  |  |  |  |  |  |  |
| 8 | 8 | SHA3/CBRD x Naxos | 181 | RIL | adult plant | natural/artificial infection | 5 | 4 | CIM | [75] |
|  |  |  |  |  |  |  |  |  |  |  |
| 5 | 4 | Chinese Spring x 8.1 | 98 | F2 | seedling stage and adult plant | natural/artificial infection | 1 | 1 | SIM | [73] |

NA, not available; RIL, recombinant inbred line; DH, double haploid; SIM, simple interval mapping; CIM, composite interval mapping; MIM, multiple interval mapping

**References**

11. Zhang KP, Zhao L, Hai Y, Chen GF, Tian JC. **QTL mapping for adult-plant resistance to powdery mildew, lodging resistance and internode length below spike in wheat.** *Acta Agron Sin* 2008, **34**:1350-1357.

12. Lan C, Ni X, Yan J, Zhang Y, Xia X, Chen X, He Z: **Quantitative trait loci mapping of adult-plant resistance to powdery mildew in Chinese wheat cultivar Lumai 21.** *Mol Breed* 2010, **25**:615-622.

55. Lan CX, Liang SS, Wang ZL, Yan J, Zhang Y, Xia XC, He ZH: **Quantitative trait loci mapping for adult-plant resistance to powdery mildew in Chinese wheat cultivar Bainong 64.** *Phytopathol* 2009, **99**:1121-1126.

56. Chen Y, Hunger RM, Carver BF, Zhang H, Yan L: **Genetic characterization of powdery mildew resistance in U.S. hard winter wheat.** *Mol Breed* 2009, **24**:141-152.

72. Jakobson I, Timofejeva HPL, Järve K: **Adult plant and seedling resistance to powdery mildew in a *Triticum aestivum* x *Triticum militinae* hybrid line.** *Theor Appl Genet* 2006, **112**:760-769.

73. Jakobson I, Reis D, Tiidema A, Peusha H, Timofejeva L, Valárik M, Kladivová M, H Šimková, Doležel J, Järve K: **Fine mapping, phenotypic characterization and validation of non-race-specific resistance to powdery mildew in a wheat–*Triticum militinae* introgression line.** *Theor Appl Genet* 2012, **125**:609-623.

74. Bougot Y, Lemoine J, Pavoine MT, Guyomar’ch H, Gautier V, Muranty H, Barloy D: **A major QTL effect controlling resistance to powdery mildew in winter wheat at the adult plant stage.** *Plant Breed* 2006, **125**:550-556.

75. [Lu](http://link.springer.com/search?facet-author=%22Qiongxian+Lu%22) Q, [Bjørnstad](http://link.springer.com/search?facet-author=%22%C3%85smund+Bj%C3%B8rnstad%22) Å, [Ren](http://link.springer.com/search?facet-author=%22Yan+Ren%22) Y, [Asad](http://link.springer.com/search?facet-author=%22Muhammad+Azeem+Asad%22) MA, [Xia](http://link.springer.com/search?facet-author=%22Xianchun+Xia%22) X, [Chen](http://link.springer.com/search?facet-author=%22Xinmin+Chen%22) X, [Ji](http://link.springer.com/search?facet-author=%22Fang+Ji%22) F, [Shi](http://link.springer.com/search?facet-author=%22Jianrong+Shi%22) J, [Lillemo](http://link.springer.com/search?facet-author=%22Morten+Lillemo%22) M: **Partial resistance to powdery mildew in German spring wheat ‘Naxos’ is based on multiple genes with stable effects in diverse environments.** *Theor Appl Genet* 2012, **125**:297-309.

76. Chantret N, Mingeot D, Sourdille P, Bernard M, Jacquemin JM, Doussinault G: **A major QTL for powdery mildew resistance is stable over time and at two development stages in winter wheat.** *Theor Appl Genet* 2001, **103**:962-971.

77. Keller M, Keller B, Schachermayr G, Winzeler M, Schmid JE, Stamp P, Messmer MM: **Quantitative trait loci for resistance against powdery mildew in a segregating wheat x spelt population.** *Theor Appl Genet* 1999, **98**:903-912.

78. Liang SS, Suenaga K, He ZH, Wang ZL, Liu HY, Wang DS, Singh RP, Sourdille P, Xia XC: **Quantitative trait loci mapping for adult-plant resistance to powdery mildew in bread wheat.** *Phytopathol* 2006, **96**:784-789.

79. Lillemo M, Asalf B, Singh RP, Huerta-Espino J, Chen XM, He ZH, Bjørnstad Å: **The adult plant rust resistance loci *Lr34*/*Yr18* and *Lr46*/*Yr29* are important determinants of partial resistance to powdery mildew in bread wheat line Saar.** *Theor Appl Genet* 2008, **116**:1155-1166.

80. Liu SX, Griffey CA, Maroof MAS: **Identification of molecular markers associated with adult plant resistance to powdery mildew in common wheat cultivar Massey.** *Crop Sci* 2001, **41**:1268-1275.

81. Mohler V, Bauer A, Bauer C, Flath K, Schweizer G, Hartl L: **Genetic analysis of powdery mildew resistance in German winter wheat cultivar Cortez.** *Plant Breed* 2011, **130**:35-40.

82. Muranty H, Pavoine MT, Jaudeau B, Radek W, Doussinault G, Barloy D: **A quantitative approach detects three QTLs involved in powdery mildew resistance at the seedling stage in the winter wheat line RE714.** *Aust J Agric Res* 2008, **59**:714-722.

83. Muranty H, Pavoine MT, Jaudeau B, Radek W, Doussinault G, Barloy D: **Two stable QTL involved in adult plant resistance to powdery mildew in the winter wheat line RE714 are expressed at different times along the growing season.** *Mol Breed* 2009, **23**:445-461.

84. Mingeot D, Chantret N, Baret PV, Dekeyser A, Boukhatem N, Sourdille P, Doussinault G, Jacquemin JM: **Mapping QTL involved in adult plant resistance to powdery mildew in the winter wheat line RE714 in two susceptible genetic backgrounds.** *Plant Breed* 2002, **121**:133-140.

85. Tucker DM, Griffey CA, Liu S, Brown-Guedira G, Marshall DS, Saghai Maroof MA: **Confirmation of three quantitative trait loci conferring adult plant resistance to powdery mildew in two winter wheat populations.** *Euphytica* 2007, **155**:1-13.

86. Wang HZ, Zhang Z, He Y, Yue JY: **Dissection and QTL mapping of component traits of resistance to wheat powdery mildew at early infection stage.** *Acta Agron Sin* 2011, **37**:1219-1228.

87. Chhuneja P, Kumar K, Stirnweis D, Hurni S, Keller B, Dhaliwal HS, Singh K: **Identification and mapping of two powdery mildew resistance genes in *Triticum boeoticum* L.** *Theor Appl Genet* 2011, **124**:1051-1058.
