# Supplementary material for: Genetic basis of qualitative and quantitative resistance to powdery mildew in wheat: from consensus regions to candidate genes
Source: BMC Genomics. 2013 Aug 19;14:562. doi: 10.1186/1471-2164-14-562 (PMC3765315; doi:10.1186/1471-2164-14-562)
Supplement: Additional file 7 — List of the Pm genes projected onto the integrated map. [file 1471-2164-14-562-S7.docx]

**Additional File 6.** List of the *Pm* genes projected onto the integrated map.

| **Gene** | **Chromosome** | **Population** | **Source** | **Reference** |
| --- | --- | --- | --- | --- |
| Pm3g | 1A | RE9001 x Courtot | *T.aestivum* | [74] |
| Pm3e | 1A | Atlantis x Cortez | *T. aestivum* | [81] |
| Mlar | 1A | Courtot x CS | *T. aestivum* | [88] |
| Pm3a | 1A | NA | NA | [56] |
| Pm24 | 1DS | CS x Chiyacao | *T. aestivum* | [89] |
| Pm4d | 2A | CS x Tm27d2 | *T. monococcum* | [53] |
| Pm23 (Pm4c) | 2AL | Line 81-7241 x Chancellor | *T. aestivum* | [90] |
| Pm4b | 2AL | RE714 x Hardi; RE714 x Festin | *T. dicoccum* | [84] |
| PmHNK54 | 2AL | Zheng 9754 x Chinese Spring | *Secale cereale* | [91] |
| MlIW70 | 2B | IW170 x 81086A | *T. dicoccoides* | [92] |
| MlZec1 | 2BL | Chinese Spring × Zecoi-1 | *T. dicoccoides* | [93] |
| PmJM22 | 2BL | Jimai 22 x Chinese Spring | *T. aestivum* | [94] |
| PmPS5B (Pm33) | 2BL | Am9/3 × Laizhou953 | *T. carthlicum* | [95] |
| Pm6 | 2BL | Am9/3 × Laizhou954 | *T. carthlicum* | [95] |
| MlAB10 | 2BL | NC97BGTAB10 x Saluda | *T. dicoccoides* | [96] |
| Pm42 | 2BS | P63 x Xuezao | *T. dicoccoides* | [97] |
| Pm43 | 2DL | CH5025 x CH5065 | *Th. intermedium* | [98] |
| Pm41 | 3BL | Langdon x IW2 | *T. dicoccoides* | [99] |
| Pm2026 | 5A | TA2026 x M389 | *T. monococcum* | [100] |
| Pm36 | 5BL | MG29896 x Latino | *T. dicoccoides* | [58] |
| Ml3D232 | 5BL | I222 x 87-1 | *T. dicoccoides* | [3] |
| Pm16 | 5BS | Chancellor x 70281 | *T. dicoccoides* | [101] |
| PmD57-5D | 5D | D57 x Yangmai 158 | *T. aestivum* | [102] |
| Pm34 | 5DL | NC97BGTD7 x Saluda | *Ae. Tauschii* | [103] |
| Pm35 | 5DL | NC96BGTD3 X Saluda | *Ae. Tauschii* | [104] |
| PmY201 | 5DL | Y2272 × Y201 | *Aegilops tauschii* | [105] |
| PmY212 | 5DL | Y2263 × Y212 | *Aegilops tauschii* | [105] |
| MlRE | 6AL | RE714 x Hardi | *T. dicoccum* | [106] |
| Pm12 | 6B | Trans. Line 31/3 x Jin 207 | *Ae. spelotides* | [107] |
| Pm27 | 6B | line 146-155 x T. timopheevii | *T. timopheevii* | [108] |
| PmG3M | 6B | Langdon x G-305-3M | *T. dicoccoides* | [109] |
| PmD57 (Pm45) | 6DS | D57 x Yangmai 158 | *T. aestivum* | [102] |
| MIAG12 | 7A | NC06BGTAG12 x Saluda | *T. timopheevii* | [110] |
| Pm37 | 7A | NCAG11 X Axminser | *T. timopheevii* | [111] |
| PmNCAG11 | 7A | Saluda x NCAG11 | *T. timopheevii* | [112] |
| PmNCA4 | 7A | Saluda x NCA4 | *T.monococcum* | [112] |
| Mlm80 | 7A | M80 x TA2025 | *T. monococcum* | [113] |
| Mlm2033 | 7A | TA2033 x TA2716 | *T. monococcum* | [113] |
| PmG16 | 7AL | Langdon x G18-16 | *T. dicoccoides* | [114] |
| MlIW72 | 7AL | IW72 x MO75 | *T. dicoccoides* | [115] |
| NCA6Pm | 7AL | NCA6 x Saluda | *T. monococcum* | [116] |
| Pm1a | 7AL | Chancellor x Axminister/8cc | *T. aestivum* | [52] |
| PmU | 7AL | CS x UR206 | *T. urartu* | [117] |
| Pm22(Pm1e) | 7AL | Chinese Spring x Virest | *T. aestivum* | [118] |
| MlRD30 | 7AL | Chinese Spring x TA2682c | *T. aestivum* | [119] |
| PmTm4 | 7BL | Tangmai 4 x Clement | *Secale cereale* L. | [120] |
| Pm5e | 7BL | Nongda 15 x Fuzhuang 30 | *T. aestivum* | [121] |
| Pm5d | 7BL | IGV1-455 x CS | *T. aestivum* | [122] |
| mlxbd | 7BL | Xiaobaidong x Chancellor | *T. aestivum* | [123] |
| Pm40 | 7BS | MY11 x GRY19 | *Elytrigia intermedium* | [124] |
| Lr34/Yr18/Pm | 7D | Thatcher x Thatcher IL | *T.aestivum* | [125] |

NA, not available

**References**

3. Zhang H, Guan H, Li J, Zhu J, Xie C, Zhou Y, Duan X, Yang T, Sun Q, Liu Z. **Genetic and comparative genomics mapping reveals that a powdery mildew resistance gene *Ml3D232* originating from wild emmer co-segregates with an NBS-LRR analog in common wheat (*Triticum aestivum* L.).** *Theor Appl Genet* 2010, **121**:1613-1621.

52. Neu C, Stein N, Keller B: **Genetic mapping of the *Lr20–Pm1* resistance locus reveals suppressed recombination on chromosome arm 7AL in hexaploid wheat.** *Genome* 2002, **45**:737-744.

53. Schmolke M, Mohler V, Hartl L, Zeller FJ, Hsam SLK: **A new powdery mildew resistance allele at the *Pm4* wheat locus transferred from einkorn (*Triticum monococcum*).** *Mol Breed* 2012, **29**:449-456.

56. Chen Y, Hunger RM, Carver BF, Zhang H, Yan L: **Genetic characterization of powdery mildew resistance in U.S. hard winter wheat.** *Mol Breed* 2009, **24**:141-152.

58. Blanco A, Gadaleta A, Cenci A, Carluccio AV, Abdelbacki AMM, Simeone R: **Molecular mapping of the novel powdery mildew resistance gene *Pm36* introgressed from *Triticum turgidum* var. *dicoccoides* in durum wheat.** *Theor Appl Genet* 2008, **117**:135-142.

74. Bougot Y, Lemoine J, Pavoine MT, Guyomar’ch H, Gautier V, Muranty H, Barloy D: **A major QTL effect controlling resistance to powdery mildew in winter wheat at the adult plant stage.** *Plant Breed* 2006, **125**:550-556.

81. Mohler V, Bauer A, Bauer C, Flath K, Schweizer G, Hartl L: **Genetic analysis of powdery mildew resistance in German winter wheat cultivar Cortez.** *Plant Breed* 2011, **130**:35-40.

84. Mingeot D, Chantret N, Baret PV, Dekeyser A, Boukhatem N, Sourdille P, Doussinault G, Jacquemin JM: **Mapping QTL involved in adult plant resistance to powdery mildew in the winter wheat line RE714 in two susceptible genetic backgrounds.** *Plant Breed* 2002, **121**:133-140.

88. Sourdille P, Robe P, Tixier MH, Doussinault G, Pavoine MT, Bernard M: **Location of *Pm3g*, a powdery mildew resistance allele in wheat, by using a monosomic analysis and by identifying associated molecular markers.** *Euphytica* 1999, **110**:193-198.

89. Huang XQ, Hsam SLK, Zeller FJ, Wenzel G, Mohler V: **Molecular mapping of the wheat powdery mildew resistance gene *Pm24* and marker validation for molecular breeding.** *Theor Appl Genet* 2000, **101**:407-414.

90. Hao Y, Liu A, Wang Y, Feng D, Gao J, Li X, Liu S, Wang H: ***Pm23*: a new allele of *Pm4* located on chromosome 2AL in wheat.** *Theor Appl Genet* 2008, **117**:1205-1212.

91. Xu W, Li C, Hu L, Wang H, Dong H, Zhang J, Zan X: **Identification and molecular mapping of *PmHNK54*: a novel powdery mildew resistance gene in common wheat.** *Plant Breed* 2011, **130**:603-607.

92. Liu Z, Zhu J, Cui Y, Liang Y, Wu H, Song W, Liu Q, Yang T, Sun Q, Liu Z: **Identification and comparative mapping of a powdery mildew resistance gene derived from wild emmer (*Triticum turgidum* var. *dicoccoides*) on chromosome 2BS.** *Theor Appl Genet* 2011, **124:**1041-1049.

93. Mohler V, Zeller FJ, Wenzel G, Hsamet SLK: **Chromosomal location of genes for resistance to powdery mildew in common wheat (*Triticum aestivum* L. em Thell.). 9. Gene *MlZec1* from the *Triticum dicoccoides*-derived wheat line Zecoi-1.** *Euphytica* 2005, **142**:161-167.

94. Yin GH, Li GY, He ZH, Liu JJ, Wang H, Xia XC: **Molecular mapping of powdery mildew resistance gene in wheat cultivar Jimai 22.** *Acta Agron Sin* 2009, **35**:1425-1431.

95. Zhu Z, Zhou R, Kong X, Dong Y, Jia J: **Microsatellite markers linked to 2 powdery mildew resistance genes introgressed from *Triticum carthlicum* accession PS5 into common wheat.** *Genome* 2005, **48**:585-590.

96. Maxwell JJ, Lyerly JH, Srnic G, Parks R, Cowger C, Marshall D, Brown-Guedira G, Murphy JP: **MlAB10: a *Triticum turgidum* subsp. *dicoccoides* derived powdery mildew resistance gene identified in common wheat.** *Crop Sci* 2010, **50**:2261-2267.

97. Hua W, Liu Z, Zhu J, Xie C, Yang T, Zhou Y, Duan X, Sun Q, Liu Z: **Identification and genetic mapping of *pm42*, a new recessive wheat powdery mildew resistance gene derived from wild emmer (*Triticum turgidum* var. *dicoccoides*).** *Theor Appl Genet* 2009, **119**:223-230.

98. He R, Chang Z, Yang Z, Yuan Z, Zhan H, Zhang X, Liu J: **Inheritance and mapping of powdery mildew resistance gene *Pm43* introgressed from *Thinopyrum intermedium* into wheat.** *Theor Appl Genet* 2009, **118**:1173-1180.

99. Li G, Fang T, Zhang H, Xie C, Li H, Yang T, Nevo E, Fahima T, Sun Q, Liu Z: **Molecular identification of a new powdery mildew resistance gene *Pm41* on chromosome 3BL derived from wild emmer (*Triticum turgidum* var. *dicoccoides*).** *Theor Appl Genet* 2009, **119**:531-539.

100. Xu H, Yao G, Xiong L, Yang L, Jiang Y, Fu B, Zhao W, Zhang Z, Zhang C, Ma Z: **Identification and mapping of *pm2026*: a recessive powdery mildew resistance gene in an einkorn (*Triticum monococcum* L.) accession.** *Theor Appl Genet* 2008, **117**:471-477.

101. Chen XM, Luo H, Xia XC, Xia LQ, Chen X, Ren ZL, He ZH, Jia ZJ: **Chromosomal location of powdery mildew resistance gene *PmI6* in wheat using SSR marker analysis.** *Plant Breed* 2005, **124**:225-228.

102. Ma H, Kong Z, Fu B, Li N, Zhang L, Jia H, Ma Z: **Identification and mapping of a new powdery mildew resistance gene on chromosome 6D of common wheat.** *Theor Appl Genet* 2011, **123**:1099-1106.

103. Miranda LM, Murphy JP, Leath S, Marshall DS: ***Pm34*: A new powdery mildew resistance gene transferred from *Aegilops tauschii* Coss. to common wheat (*Triticum aestivum* L.).** *Theor Appl Genet* 2006, **113**:1497-1504.

104. Miranda LM,. Murphy JP, Marshall D, Cowger C, Leath S: **Chromosomal location of *Pm35*, a novel *Aegilops tauschii* derived powdery mildew resistance gene introgressed into common wheat (*Triticum aestivum* L.).** *Theor Appl Genet* 2007, **114**:1451-1456.

105. Sun XL, Liu D, Zhang HQ, Huo NX, Zhou RH, Jia JZ: **Identification and mapping of two new genes conferring resistance to powdery mildew from *Aegilops tauschii* (Coss.) Schmal.** *J Integr Plant Biol* 2006, **48**:1204-1209.

106. Chantret N, Sourdille P, Röder M, Tavaud M, Bernard M, Doussinault G: **Location and mapping of the powdery mildew resistance gene MlRE and detection of a resistance QTL by bulked segregant analysis (BSA) with microsatellites in wheat.** *Theor Appl Genet* 2000, **100**:1217-1224.

107. Song W, Xie H, Liu Q, Xie C, Ni Z, Yang T, Sun Q, Liu Z: **Molecular identification of *Pm12*-carrying introgression lines in wheat using genomic and EST-SSR markers.** Euphytica 2007, **158**:95–102.

108. Jarve K, Peusha HO, Tsymbalova J, Tamm S, Devos KM, Enno TM: **Chromosomal location of a *Triticum timopheevii* - derived powdery mildew resistance gene transferred to common wheat.** *Genome* 2000, **43**:377-381.

109. Xie W, Ben-David R, Zeng B, Distelfeld A, Roder MS, Dinoor A, Fahima T: **Identification and characterization of a novel powdery mildew resistance gene *PmG3M* derived from wild emmer wheat, *Triticum dicoccoides*.** *Theor Appl Genet* 2011, **124**:911-922.

110. Maxwell JJ, Lyerly JH, Cowger C, Marshall D, Brown-Guedira G, Murphy JP: **MlAG12: a *Triticum timopheevii*-derived powdery mildew resistance gene in common wheat on chromosome 7AL.** *Theor Appl Genet* 2009, **119**:1489-1495.

111. Perugini LD, Murphy JP, Marshall D, Brown-Guedira G: ***Pm37*, a new broadly effective powdery mildew resistance gene from *Triticum timopheevii*.** *Theor Appl Genet* 2008, **116**:417-425.

112. Srnic´ G, Murphy JP, Lyerly JH, Leath S, Marshall DS: **Inheritance and chromosomal assignment of powdery mildew resistance genes in two winter wheat germplasm lines.** *Crop Sci* 2005, **45**:1578-1586.

113. Yao G, Zhang J, Yang L, Xu H, Jiang Y, Xiong L, Zhang C, Zhang Z, Ma Z, Sorrells M E:**Genetic mapping of two powdery mildew resistance genes in einkorn (*Triticum monococcum* L.) accessions.** *Theor Appl Genet* 2007, **114**:351-358.

114. Ben-David R, Xie W, Peleg Z, Saranga Y, Dinoor A, Fahima T: **Identification and mapping of *PmG16*, a powdery mildew resistance gene derived from wild emmer wheat.** *Theor Appl Genet* 2010, **121**:499-510.

115. Ji X, Xie C, Ni Z, Yang T, Nevo E, Fahima T, Liu Z, Sun Q: **Identification and genetic mapping of a powdery mildew resistance gene in wild emmer (*Triticum dicoccoides*) accession IW72 from Israel.** *Euphytica* 2008, **159**:385-390.

116. Miranda LM, Perugini L, Srnic’ G, Brown-Guedira G, Marshall D, Leath S, Murphy JP: **Genetic mapping of a *Triticum monococcum* derived powdery mildew resistance gene in common wheat.** *Crop Sci* 2007, **47**:2323-2329.

117. Qiu YC, Zhou RH, Kong XY, Zhang SS, Jia JZ **Microsatellite mapping of a *Triticum urartu* Tum. derived powdery mildew resistance gene transferred to common wheat (*Triticum aestivum* L.).** *Theor Appl Genet* 2005, **111**:1524-1531.

118. Singrun Ch, Hsam SLK, Hartl L, Zeller FJ, Mohler V: **Powdery mildew resistance gene *Pm22* in cultivar Virest is a member of the complex *Pm1* locus in common wheat (*Triticum aestivum* L. em Thell.).** *Theor Appl Genet* 2003, **106**:1420-1424.

119. Singrun Ch, Hsam SLK, Zeller FJ, Wenzel G, Mohler V: **Localization of a novel recessive powdery mildew resistance gene from common wheat line RD30 in the terminal region of chromosome 7AL.** *Theor Appl Genet* 2004, **109**:210-214.

120. Hu TZ, Li HJ, Xie CJ, You MS, Yang ZM, Sun QX, Liu ZY: **Molecular mapping and chromosomal location of the powdery mildew resistance gene in wheat cultivar tangmai 4.** *Acta Agron Sin* 2008, **34**:1193-1198.

121. Huang XQ, Wang LX, Xu MX, Röder MS: **Microsatellite mapping of the powdery mildew resistance gene *Pm5* e in common wheat (*Triticum aestivum* L.).** *Theor Appl Genet* 2003, **106**:858-865.

122. Nematollahi G, Mohler V, Wenzel G, Zeller FJ, Hsam SLK: **Microsatellite mapping of powdery mildew resistance allele *Pm5d* from common wheat line IGV1-455.** *Euphytica* 2008, **159**:307-313.

123. Xue F, Zhai WW, Duan XY, Zhou YL, Ji WQ: **Microsatellite mapping of powdery mildew resistance gene in wheat landrace Xiaobaidong.** *Acta Agron Sin* 2009, **35**:1806-1811.

124. Luo PG, Luo HY, Chang Z J, Zhang HY, Zhang M, Ren ZL: **Characterization and chromosomal location of *Pm40* in common wheat: a new gene for resistance to powdery mildew derived from *Elytrigia intermedium*.** *Theor Appl Genet* 2009, **118**:1059-1064.

125. Spielmeyer W, McIntosh RA, Kolmer J, Lagudah ES: **Powdery mildew resistance and *Lr34* / *Yr18* genes for durable resistance to leaf and stripe rust cosegregate at a locus on the short arm of chromosome 7D of wheat.** *Theor Appl Genet* 2005, **111**:731-735.
