# Supplementary material for: Genetic basis of qualitative and quantitative resistance to powdery mildew in wheat: from consensus regions to candidate genes
Source: BMC Genomics. 2013 Aug 19;14:562. doi: 10.1186/1471-2164-14-562 (PMC3765315; doi:10.1186/1471-2164-14-562)
Supplement: Additional file 8 — Co-localization of the QTL and MQTL with disease-resistance-related genes. [file 1471-2164-14-562-S8.docx]

**Additional File 7.** Co-localization of the QTL and MQTL with disease-resistance-related genes.

There are two markers with putative functions of interest under MQTL1 on chromosome 1A: the marker *wPt-1862* (45.1 cM), which corresponds to a NBS-LRR protein and has 44% identity at the amino-acid level with the *Pm3* gene; and the marker *Xcdo1160* (53.6 cM), which corresponds to a putative ketol-acid reductoisomerase, an enzyme induced by plant–pathogen interactions [54]. Another marker putatively corresponding to the same enzyme is *Xmwg632*, which maps at 80.3 cM, under the individual QTL QPm_Lan [55]. The DArT marker *wPt-0432* (84 cM), which corresponds to a NBS-LRR protein, is positioned a few cM from the individual QTL QPm.osu-1A [56]. With the three DArT markers, *wPt-1912* (15 cM), *wPt-4107* (16.1 cM), and *wPt-1560* (16.5 cM), the first two putatively correspond to NBS-LRR proteins, and the third to a protein kinase; these are mapped within the region of MQTL3 on chromosome 1B. The marker *wPt-1912*, in particular, shows 69% identity at the amino-acid level with the *R* gene *Lr21* of *T. aestivum*. Three other genes of interest include a thioredoxin, 1,4-benzoquinone reductase, and a protein kinase (markers *Swes578* - 51.6 cM, *CA651264* - 55.9 cM, *wPt-5011* 59.1 cM*,* respectively), and these are positioned under the individual QTL QPm.osu-1B [56].

The DArT marker *wPt-5865*, which putatively codes for a NBS-LRR protein, is mapped at 69.4 cM on chromosome 2A, together with the resistance gene *PmHNK54*, under MQTL4. At a very short distance away (4.7 cM) there is the DArT marker *wPt-7056*, which corresponds to a protein kinase. Finally, two other NBS-LRR proteins are positioned within the region of two individual QTL (QPm.inra.2A, QPm_sugg), with the markers *wPt-6064* (80.3 cM) and *wPt-7024* (78.5 cM); the second of these, in particular, co-mapped with the resistance gene *Pm23*. The markers *wPt-9257* - 130.7 cM - (LRR receptor kinase), *wPt-9644* - 69.6 cM - (protein kinase), *Xmag464* - 69.6 cM - (heat shock factor), and *wPt-2600* - 69.8 cM - (NBS-LRR protein) share the same genetic position (within 4 cM) with the resistance gene *Pm42* on chromosome 2B. The markers *BJ253815* - 122.2 cM - (metallothionein), *Xcdo244* - 129.4 cM - (NBS-LRR protein), and *wPt-9257* - 130.7 cM - (LRR-kinase protein) fall into the region covered by MQTL8 on the long arm of chromosome 2B, together with the resistance gene *PmJM22*. A number of other markers that putatively correspond to genes of interest are positioned within regions covered by individual QTL on the same chromosome: e.g., *wPt-2120* (73.3 cM), *wPt-0950* (93 cM), *wPt-0189* (113.3 cM), and *wPt-4368* (116.7 cM), which represent *NBS-LRR* genes; and *Xbcd292* - 106.2 cM - (peroxisome biogenesis protein 22-like), *wPt-1650* - 106.4 cM - (Ca-binding protein), and *wPt-1646* - 119.7 cM - (protein kinase). Finally, the DArT marker *wPt-6752* (NBS-LRR) is positioned at 3.1 cM from the resistance gene *Pm43*, on chromosome 2D.

Eight markers that correspond to genes of interest are located near MQTL10 on chromosome 3A: *Xbcd22* - 27.2 cM - (glycosyl transferase family 8); *wPt-9049* - 42.4 cM and *wPt-2698* - 44.7 cM - (PHD zinc finger protein-like); *BJ213673c* - 45 cM - (CTD-phosphatase-like protein); *wPt-2938* - 48.4 cM - (WRKY transcription factor 30); *Xbcd828* - 50.1 cM - (H^+^-transporting ATP synthase beta chain); *wPt-0714* - 52.1 cM - (HEAT repeat family protein); and *Xmag620b* - 52.4 cM - (zinc finger, C2H2 type). The marker *TC74823a* - 64.8 cM -(SGNH_hydrolase) is within MQTL10. Two other markers (*wPt-6422* - 76.7 cM, a putative transcription factor X1, and *wPt-4077* - 82.5 cM, a NBS-LRR protein) are in the single QTL QPm_RF. The marker *Xbcd372*, which corresponds to glycosyltransferase family 8B, is positioned at 2 cM from *wPt-4077*. Moreover, the putative pathogenesis-related protein 1-12 (*TC77302* - 151.8 cM) maps in the telomeric region of the chromosome. The DArT markers *wPt-5697* - 6 cM - (protein kinase) and *wPt-1516* - 11.7 cM - (NBS-LRR protein) are under QTL CP 2, on chromosome 3B.

The DArT marker *wPt-6303* - 55.6 cM - (NBS-LRR protein) is positioned 1 cM away from the region corresponding to MQTL11, on chromosome 4A. Four other DArT markers that putatively code for NBS-LRR proteins fall within the region of MQTL13, on the same chromosome. One of these, *wPt-3729* (177.6 cM), shows 83% identity with the rust resistance gene Rp1-like of *T. aestivum*. Markers of the same type that map under individual QTL are also observed: wPt*-7491a* - 154.4 cM - (protein kinase), *Xmag974* - 155.9 cM - (glutathione transferase), and *wPt-9183* - 157.2 cM , *wPt-4241* - 162.4 cM, *wPt-5172* - 167 cM, *wPt-2951* - 179.6 cM, and *wPt-0150* - 180.6 cM (NBS-LRR proteins).

Some genes of interest are positioned in regions that correspond to individual QTL on chromosome 5A: a serine/ threonine-protein kinase Nek2-like (*TC91851* - 85.4 cM); a protein kinase (*Xmag1159* - 102.6 cM); a heat shock protein (*Xcdo412* - 103.4 cM); and a NBS-LRR protein (*wPt-9800a* - 174 cM). For chromosome 5B, the marker *wPt-3569* (109.1 cM), which corresponds to a protein kinase, falls into the region of MQTL17, while the marker *wPt-8604* - 74.3 cM - (LRR receptor-like protein kinase) maps under the individual QTL QTL-5B, nearly 9 cM from the resistance gene *Pm16*.

The marker *wPt-7655* (130.4 cM) putatively corresponds to a 1,3-beta-glucan synthase component and is positioned under MQTL20, on chromosome 6A. Three other markers fall into the CI of the individual QTL QPm98F2: *wPt-8331* - 89.2 cM and *wPt-3191a* - 114.6 cM (NBS-LRR proteins); and *Xcdo836* - 137 cM (cytosolic glutathione reductase). The DArT marker *wPt-7445* - 62.6 cM (NBS-LRR protein) is included in the region of the QTL CP 1 on the same chromosome. Two markers, *wPt-3191b* - 98.2 cM (NBS-LRR protein) and *wPt-9881* - 99.5 cM (putative acyl-CoA-binding protein) are positioned under the individual QTL PMm, on chromosome 6B [75].

There are many genes of interest associated to individual QTL on chromosome 7A. The markers *wPt-4487b* - 36.2 cM - and *wPt-7491b* - 43.1 cM (LRR protein kinases), *wPt-6966* - 36.6 cM, *wPt-3648* - 41 cM - and *wPt-3434* - 43.6 cM - (NBS-LRR proteins), and *wPt-1441* - 37.9 cM (acyl-protein synthetase) are positioned under ‘QTL-7A’ on the short arm. The marker *wPt-6966*, in particular, shows 49% identity at the amino-acid level to the *B. distachyon* gene *Lr21* for resistance to leaf rust. The DArT marker *wPt-1080* - 93.7 cM - (protein kinase) is under the QTL PMm, and *Xcdo673* - 154.8 cM - (LRR protein kinase) is under the QTL QPm97F2. Markers *wPt-3403* - 204.6 cM - (NBS-LRR protein) and *Xmag1759* - 206.6 cM - (protein kinase) are in a small region comprised between the resistance genes *PmNCAG11* and *Mlm80* under the individual QTL QPm.inra-7A3-b and QPm98F2. Finally, the marker *Xcdo347* - 234.6 cM - (callose synthase-like protein) co-maps with the gene *Pm1* at 5 cM from *TC92445* (pathogenesis-related protein 1-15) under the QTL QPm.inra-7A3a. The marker *wPt-7925* - 81.7 cM - (LRR receptor-like protein kinase) is positioned at 0.2 cM from the resistance gene *Pm40* under the individual QTL QPmV.inra.7B, on chromosome 7B. The markers *wPt-8417* - 123.6 cM - (S-adenosylmethionine-dependent methyltransferase), *wPt-4342* - 124.8 cM - and *wPt-0194* - 126.6 cM - (NBS-LRR proteins) are mapped in the chromosomal region shared by the individual QTL QPm.2_K and QPm_RF. The marker *wPt-6701* - 147.3 cM - (protein kinase) mapped 2 cM away from the resistance gene *Pm5e* under the individual QTL QPm_L and QPm_RF. Under the last of these QTL, the markers *wPt-4902* - 160.4 cM - (putative callose synthase 1 catalytic subunit), *wPt-4875* - 161.3 cM - (protein kinase), *wPt-4319b* - 163 cM - and *wPt-9515* - 163 cM - (NBS-LRR proteins) are also mapped.

**References**

54. Freitas-Astúa J, Bastianel M, Locali-Fabris EC, Novelli VM, Silva-Pinhati AC, AC Basílio-Palmieri, Targon MLPN, Machado MA: **Differentially expressed stress-related genes in the compatible citrus-Citrus leprosis virus interaction.** *Genet Mol Biol* 2007, **30**:980-990.

55. Lan CX, Liang SS, Wang ZL, Yan J, Zhang Y, Xia XC, He ZH: **Quantitative trait loci mapping for adult-plant resistance to powdery mildew in Chinese wheat cultivar Bainong 64.** *Phytopathol* 2009, **99**:1121-1126.

56. Chen Y, Hunger RM, Carver BF, Zhang H, Yan L: **Genetic characterization of powdery mildew resistance in U.S. hard winter wheat.** *Mol Breed* 2009, **24**:141-152.

75. [Lu](http://link.springer.com/search?facet-author=%22Qiongxian+Lu%22) Q, [Bjørnstad](http://link.springer.com/search?facet-author=%22%C3%85smund+Bj%C3%B8rnstad%22) Å, [Ren](http://link.springer.com/search?facet-author=%22Yan+Ren%22) Y, [Asad](http://link.springer.com/search?facet-author=%22Muhammad+Azeem+Asad%22) MA, [Xia](http://link.springer.com/search?facet-author=%22Xianchun+Xia%22) X, [Chen](http://link.springer.com/search?facet-author=%22Xinmin+Chen%22) X, [Ji](http://link.springer.com/search?facet-author=%22Fang+Ji%22) F, [Shi](http://link.springer.com/search?facet-author=%22Jianrong+Shi%22) J, [Lillemo](http://link.springer.com/search?facet-author=%22Morten+Lillemo%22) M: **Partial resistance to powdery mildew in German spring wheat ‘Naxos’ is based on multiple genes with stable effects in diverse environments.** *Theor Appl Genet* 2012, **125**:297-309.
